# Phylogenetic signal analysis in the basicranium of Ursidae (Carnivora, Mammalia)

**DOI:** 10.7717/peerj.6597

**Published:** 2019-03-15

**Authors:** María Eugenia Arnaudo, Néstor Toledo, Leopoldo Soibelzon, Paula Bona

**Affiliations:** 1 División de Paleontología Vertebrados, Facultad de Ciencias Naturales y Museo-UNLP, La Plata, Buenos Aires, Argentina; 2 CONICET, Consejo Nacional de Investigaciones Científicas y Técnicas, Buenos Aires, Argentina; 3 División Paleontología Vertebrados, Unidades de Investigación Anexo Museo, Facultad de Ciencias Naturales y Museo-UNLP, La Plata, Buenos Aires, Argentina; 4 Laboratorio de Morfología Evolutiva y Desarrollo (MORPHOS)-División de Paleontología Vertebrados, Facultad de Ciencias Naturales y Museo-UNLP, La Plata, Buenos Aires, Argentina

**Keywords:** Basicranium, Principal component analysis, Orthonormal decomposition, Ursidae, Phylogenetic signal

## Abstract

Ursidae is a monophyletic group comprised of three subfamilies: Tremarctinae, Ursinae and Ailuropodinae, all of which have a rich geographical distribution. The phylogenetic relationships within the Ursidae group have been underexamined, especially regarding morphological traits such as the basicranium. Importantly, the basicranium is a highly complex region that covers a small portion of the skull, combining both structural and functional aspects that determine its morphology. Phylogenetic hypotheses of the Ursidae (including Tremarctinae) have been made based on morphological characters that considers skull, mandible and teeth features, while specific characters of the auditory region and basicranium have not been taken into account. To do this, we analyse the shape and size macroevolution of the basicranium of Ursidae, testing its morphological disparity in a phylogenetic context, which is quantified by means of the phylogenetic signal. We investigated phylogenetical autocorrelation by shape (depicted by Principal Components Analysis scores from previous published analyses) and basicranium size (depicted by centroid size, CS) using an orthonormal decomposition analysis and Abouheif C mean. The main advantages of these methods are that they rely exclusively on cladogram topology and do not require branch-length estimates. Also, an optimisation of the ancestral nodes was performed using TNT 1.5 software. In relation to the phylogenetic signal, both methods showed similar results: the presence of autocorrelation was detected in PC1 and PC2, while in PC3, PC4 and PC5 and in the size of the basicranium (CS), the absence of autocorrelation occurred. The most significant nodes (where there is autocorrelation) are the basal nodes ‘Ursidae’ and ‘Ursinae-Tremarctinae’. Within this last group, distinctive basicranium morphology is observed, being more conservative in Tremarctinae than in Ursinae. The differences between these subfamilies could be related to historical events involving varying food and environmental preferences. The high phylogenetic signal in the node Tremarctinae probably indicates that the basicranium configuration of these bears was obtained early in their evolutionary history. Finally, our results of the basicranium and skull length ratios indicate that in Tremarctinae, the basicranium size was not determined by phylogeny but instead by other factors, such as adaptive responses to climatic changes and competition with other carnivores.

## Introduction

Ursidae is a monophyletic group of placental carnivoran mammals comprised of three subfamilies: Ursinae, Ailuropodinae and Tremactinae. The family has been found in America, Asia, Europe, Africa and India from the late Paleogene to recent times.

Within Ursinae, three extant genera are recognised, encompassing the following species: the American black bear *Ursus americanus*, the brown bear *U. arctos*, the polar bear *U. maritimus*, the Asian black bear *U. thibetanus*, the sloth bear *Melursus ursinus,* the sun bear *Helarctos malayanus* and several fossil representatives (Wilson & Reeder, 2005; Garshelis, 2009). This subfamily is distributed throughout Eurasia, North America and—in the past—in the Atlas Mountains of North Africa (Garshelis, 2009) since the early Miocene period up to recent times (Krause et al., 2008).

The Ailuropodinae includes just one living species, the giant panda *Ailuropoda melanoleuca*, which is distributed in the mountains of the central region of China. Although its systematics have been a matter of debate, it is currently considered a member of Ursidae (O’Brien et al., 1985; Krause et al., 2008; Juárez Casillas & Varas, 2011). The oldest record of this subfamily corresponds to the late Pliocene of China and the Asian Southeast (Jin et al., 2007).

Tremarctinae is comprised of four genera: *Plionarctos*; *Arctodus*; *Arctotherium* and *Tremarctos*. *Plionartos* is comprised of *P. edenensis* and *P. harroldorum*; *Arctodus* contains *A r . pristinus* and *A r . simus*; within *Arctotherium* are the following species: *A. angustidens*, *A. vetustum*, *A. bonariense*, *A. tarijense* and *A. wingei*; and finally *Tremarctos* includes *T. floridanus* and the extant spectacled bear *T. ornatus*, being the latest species of the only extant Tremarctinae (Soibelzon, 2002; Soibelzon, 2004). This subfamily is distributed exclusively in America, from Alaska to the southern region of Chile; it was first recorded in the late Miocene in North America (Soibelzon, Tonni & Bond, 2005).

The basicranium in mammals is a complex region that forms the floor of the brain case (a relatively small portion of the ventral surface of the skull). In mammals, the brain fills the cerebral cavity by up to 95% (Jerison, 1973), so the morphology and dimensions of the brain case gives an approximation of the shape and relative size of the encephalon and main sensitive organs contained within there. The basicranium presents neurovascular foramina for the passage of several cranial nerves (i.e., CNs XII-IX, the mandibular branch of the trigeminous nerve, the cordae tympani nerve, the facial nerve VII, the auricular branch of vagus nerve, etc.) veins and arteries (i.e., the internal jugular vein, the internal carotid artery and veins from the transverse and inferior petrosal sinuses, the internal facial vein, stylomastoid artery, etc.) and the opening of the Eustachian tubes. Other bony landmarks such as muscular attachments and other anatomical structures (e.g., outline and relative size of the tympanic, hyojugular fossa) are often used in comparative anatomical studies and are related to several biological functions such as mastication, balance and audition, among others (e.g., Davis, 1964; Wible, 1986; Wible, 1987; Wozencraft, 1989; Lieberman, Ross & Ravosa, 2000; Strait, 2001). Despite some authors previously considering that the basicranium is morphologically conservative (e.g.,  Turner, 1848), it is an element of potential importance in phylogeny and hence in the evolutionary history of carnivores specifically and in mammals generally (Mitchell & Tedford, 1973; Radinsky, 1969; Radinsky, 1971; Radinsky, 1973; Radinsky, 1974; Neff, 1987; Wozencraft, 1989; Wible & Hopson, 1993; Wang & Tedford, 1994; Lieberman, Ross & Ravosa, 2000; Hunt, 2001).

Given that the braincase and basicranium reflect the morphology of the encephalon and sense organs, a study of the basicranium can provide inferences related to a mammal’s behaviour. This is particularly relevant in paleobiological analyses, when fossil specimens preserve only fragments of the brain case and when no data of the postcranium or cranio-dental traits can be recovered.

Similar to what occurs with other parts of the skeleton, the morphological variation of the brain case can be explained by a combination of phylogenetic history and autapomorphic adaptations to different life habits, as well as the animal’s ability to move and feed. In this sense, an interesting approach for interpreting the morphological variation within a lineage is the analysis of the life history traits of taxa expressed as quantitative variables in a phylogenetic context (Ollier, Couteron & Chessell, 2006). When close relatives in a phylogeny are more similar than distant relatives, the morphological pattern observed presents a phylogenetic signal (Harvey & Pagel, 1991). Species’ traits can show a high or low phylogenetic signal; when the phylogenetic signal is high, closely related species exhibit similar trait values, and trait similarity decreases as phylogenetic distance increases (Losos, 2008). Conversely, a trait that shows a weak phylogenetic signal may vary randomly across a phylogeny, and distantly related species often converge on a similar trait value, while closely related species exhibit notably different trait values (Kamilar & Muldoon, 2010; Kamilar & Cooper, 2013).

The morphological disparity of the basicranium of Ursidae has often been discussed (e.g., Torres, 1988; García et al., 2007; Rabeder, Pacher & Withalm, 2010; Santos et al., 2014; Koufos, Konidaris & Harvati, 2017; Arnaudo & Fernandez Blanco, 2016), but almost all of these studies have focused on ursine bears (e.g., *Ursus spelaeus*, *U. deningeri*). The phylogenetic hypotheses of the Ursidae (including Tremarctinae) have been made based on morphological characters that consider skull, mandible and teeth features using a molecular analysis; however, the specific characteristics of the auditory region and basicranium have not been taken into account (e.g.,  Trajano & Ferrarezzi, 1994; Ubilla & Perea, 1999; Soibelzon, 2002; Pagès et al., 2008; Soibelzon, Schubert & Posadas, 2010; Kumar et al., 2017).

The aim of the current work is to analyse the macroevolution of the shape and size of the braincase of Ursidae, testing its morphological variations in a phylogenetic context. To accomplish this, we used previous results published by Arnaudo & Fernandez Blanco (2016), who studied the auditory region and basicranium of Ursidae using a two-dimensional geometric morphometric approach (see the supplementary material for further explanation). Arnaudo & Fernandez Blanco (2016) found that the main groups of bears (i.e., subfamilies) can be clustered by the shape of the basicranium and auditory region, and the authors concluded that the disparity of that part of the brain case could be explained by the phylogenetic history of the clade.

## Materials and Methods

The cranium of Ursidae was analysed from a sample of 164 extinct and extant species, as listed in Table S1. The morphological variation of the basicranium was considered using the PC scores reported by Arnaudo & Fernandez Blanco (2016), which were based on two-dimensional geometric morphometric (Table S2 and also see the summary of the results from (Arnaudo & Fernandez Blanco, 2016) in the supplementary material for further explanation).

Because of the lack of available phylogenetic hypotheses, including the fossils of ursids (especially tremarctines), a super tree was built from two different sources using the Mesquite software package (Maddison & Maddison, 2017). For extant species, the phylogeny taken into account was proposed by Krause et al. (2008), and this was based on molecular data. An extended approach to build more complete phylogenies is to assemble super trees by combining these backbone phylogenies with smaller, overlapping trees (Bininda-Emonds, 2004; Baker et al., 2009). Because of this, super trees usually lack accurate branch-length information, or branch-length data can even be missing (i.e., the resultant super trees only provide topological information; (Molina-Venegas 2017).

**Figure 1:**
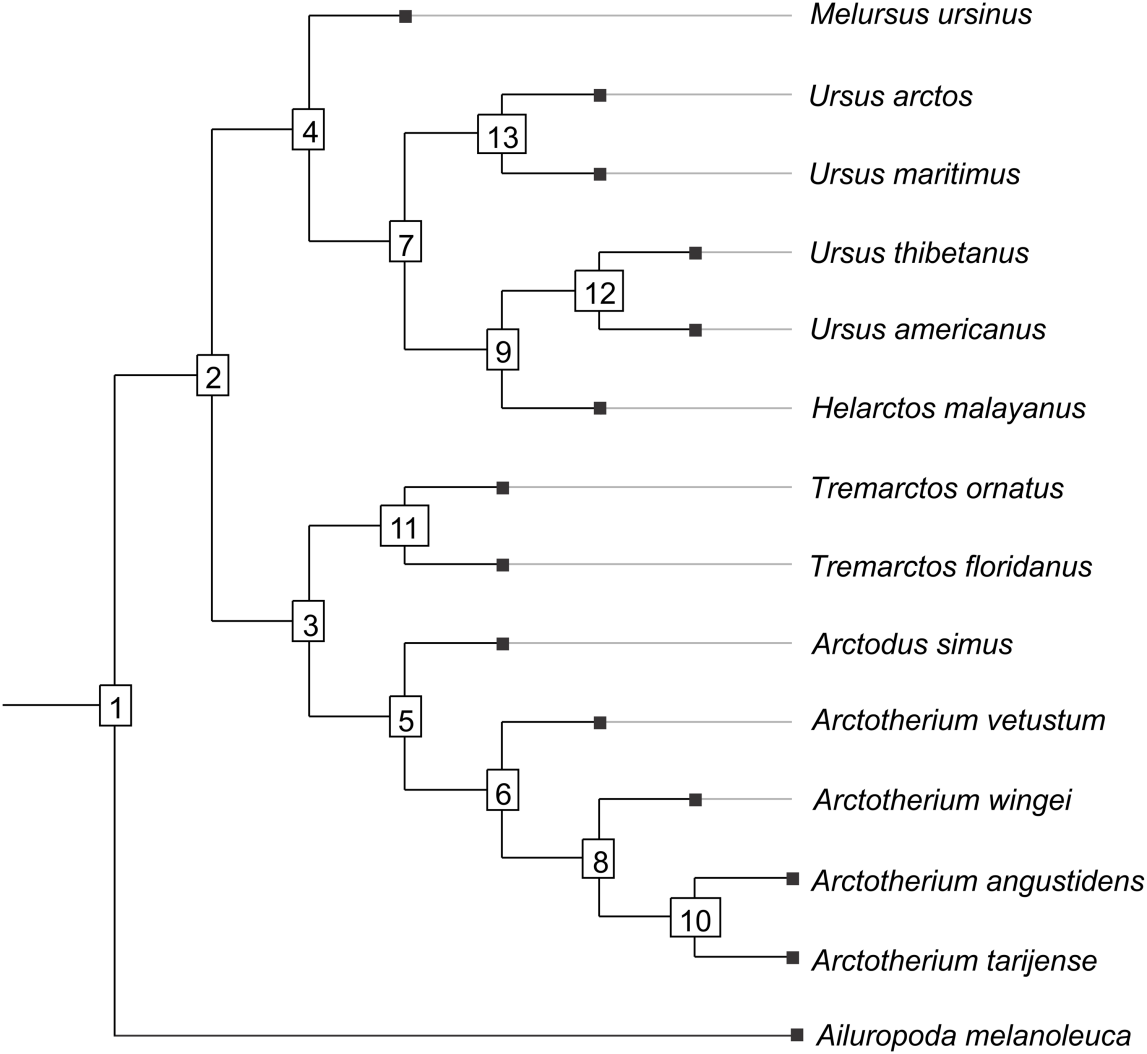
Cladogram B. Phylogenetic tree topology of the Cladogram A used in this study for the Ursidae family. The numbers of the clade correspond with the numbers obtained using orthonormal decomposition analysis.

Two different phylogenetic hypotheses were compared. The first—cladogram A (Mitchell et al., 2016)—is based on molecular data and takes *Arctodus* as a sister taxon of the clade formed by *Arctotherium* + *Tremarctos* (Fig. 1).

The second hypothesis—cladogram B (Soibelzon, 2002)—is based on morphological data and considers the spectacled bear clade (*Tremarctos floridanus* and *T. ornatus*) to be the sister group of the short-faced bear clade (which includes *Arctodus* and *Arctotherium*; Fig. S1).

### Phylogenetical autocorrelation—Orthonormal decomposition and Abouheif’s C mean methods

We performed analyses to search for phylogenetical autocorrelations in shape and size (depicted by centroid size, CS; Hood, 2000). As described in Arnaudo & Fernandez Blanco (2016), major shape variations were a focus in the first and second Principal Components (PCs) (PC1 = 48.5% and PC2 = 15.7%), with the explained variance dropping below the 5% beyond the fourth PC (Fig. S2). Thus, we analysed the phylogenetical signal present only in the first five PCs. Because the inference of branch length for the fossil taxa could not be carried out with certainty, we used two tests that did not require estimating the branch lengths, relying exclusively on cladogram topology instead: the orthonormal decomposition (Ollier, Couteron & Chessell, 2006) and the Abouheif (Abouheif, 1999) analyses. All calculations were performed in the R free statistical suite (R Core Team, 2018), employing different tools from the *ade4* (Dray & Dufour, 2007), *ape* (Paradis, Claude & Strimmer, 2004), *geiger* (Harmon et al., 2008), *adephylo* (Dray & Jombart, 2008) and *phylosignal* (Keck et al., 2016) packages.

The orthonormal decomposition analysis allows for the detection of specific nodes where autocorrelation is higher. This test (as implemented in the *ade4* package) builds a matrix of orthobases (i.e., orthonormal vectors depicting the topological information of the tree) and then analyses the correlation between the studied variables (PC scores and CS) and each orthobasis vector by means of four nonparametric statistics. The construction of a null model of no-correlation and confidence intervals (at alpha 0.05) for the statistics is achieved by Monte Carlo permutations of the orthobases vector matrix against the studied variables. The R2Max (maximal R2) depicts high values whenever a significant share of dependence is detected at a single node (otherwise, dependence is overspread through several nodes). The Dmax (maximal deviation) corresponds to the Kolmogorov–Smirnov statistic and tests if the studied variable is similar to a random sample from a uniform distribution. The SkR2k (sum of k-nth R2) depicts the skewness toward the tree’s tips or roots, that is, the proportion of variance explained by basal nodes versus terminal ones. The sum of cumulative errors (SCE) describes the averaged variation.

Abouheif’s C index is considered a special case of spatial correlation index in Moran’s I (Gittleman & Kot, 1990); it was performed for studying the correlation between the studied variables (PC scores and CS) and a matrix of phylogenetic proximities with a non-null diagonal (see Pavoine et al., 2008), which summarises the topology of the cladogram. The calculation of the proximity matrix was performed using the ‘oriAbouheif’ method of the command *proxTips* (*adephylo* R package), as discussed in Pavoine et al. (2008). Then, this matrix was used as an input for the *gearymoran* function of the *ade4* R package. The null hypothesis is the absence of correlation (the C mean equals 0), and the significance of the observed parameter is tested against a distribution built on permutations.

### Landmark optimisation

Optimisation of the ancestral nodes was performed using TNT 1.5 software (Goloboff & Catalano, 2016); this version integrates landmark data from TPS files into a phylogenetic analysis. Landmark data consist of coordinates (in two or three dimensions) for the terminal taxa; TNT reconstructs shapes for the internal nodes such that the difference between the ancestor and descendant shapes for all tree branches sums up to a minimum. Then, this sum is used as tree score (Goloboff & Catalano, 2016).

### Skull proportions

To compare relative changes in the shape of the basicranium with respect to the skull as a whole, a ratio between the anteroposterior length of the basicranium (bsL) and the anteroposterior total length of the skull (stL) is used.

This ratio describes the proportional length of the basicranium compared with the rest of the skull. Measurements were taken on orientated photographs of almost the total sample (some fossil specimens were incomplete, so the total length could not be measured for these) of the ursids using ImageJ software (Rasband, 2006). The differences between subfamilies were analysed using nonparametric statistical tests (Kruskal–Wallis and Wilcoxon rank-sum tests). Calculations were performed in R using the *kruskal.test* and *pairwise.wilcox.test* functions of the core package *stats*.

## Results

### Orthonormal decomposition of variance (Figs. S3–S15)

The results obtained rendered no differences between the phylogenetic hypotheses considered in the current study, so to avoid repetition, we describe the results concerning the cladogram A, which was recently published by Mitchell et al. (2016) (for results concerning the cladogram B, see Figs. S5, S7, S9, S11, S13 and S15).

**Figure 2:**
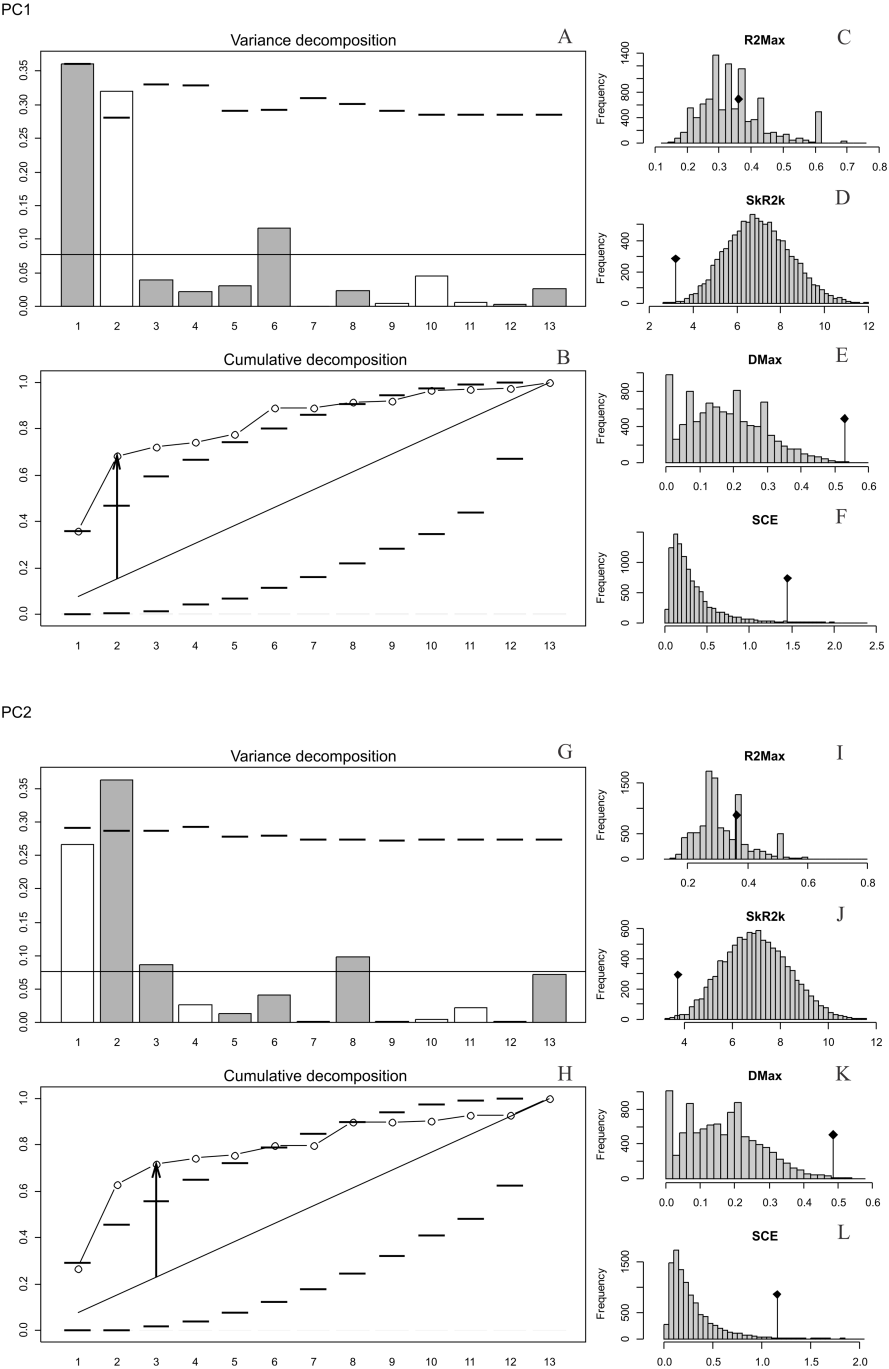
Orthonormal decomposition results of PC1 and PC2 for Cladogram A. (A, G) Orthogram plot: height of bars is proportional to the squared coefficients (white and grey bars represents positive and negative coefficients); dashed line is the upper confidence limit at 5%, built from Monte Carlo permutations; horizontal solid line is the mean value; (B, H) Cumulative orthogram plot: circles represent observed values of cumulated squared coefficients (vertical axis); the expected values under H_0_ are disposed on the straight line; dashed lines represent the bilateral confidence interval; (C–F; I–L) Histograms of observed values of the four statistic tests: black dot depicts the observed parameter value.

The presence of autocorrelation was detected in PC1 and PC2 (Figs. 2A–2L), while PC3, PC4 and PC5 and the size of the basicranium (CS) did not show any significant differences from the null model of a uniform distribution of the orthogram values (Figs. S10, S12, S14). We infer absence of a phylogenetic signal in CS, PC3, PC4 and PC5 (Figs. S4, S10, S12, S14), because the observed values of the four corresponding statistics were all exceeded by the results of many Monte Carlo randomisations (see Ollier, Couteron & Chessell, 2006). Finally, the values of the cumulated orthogram remained within the confidence limits.

**Figure 3:**
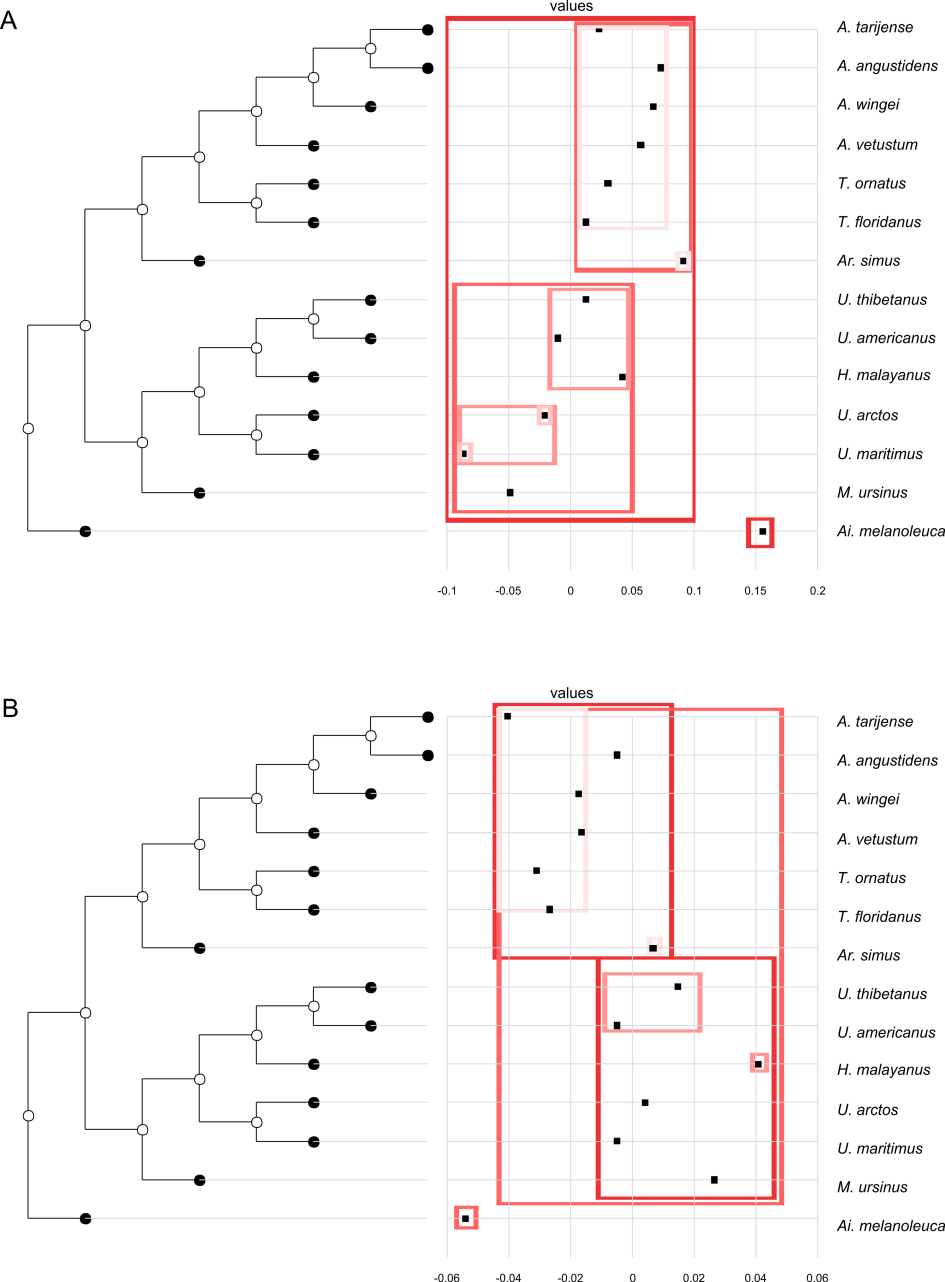
Phylogenetic tree with dotplot of the shape of the basicranium (depicted as PC scores) and species names for PC1 (A) and PC2 (B). Boxes in shades of red enclose variation explained by nodes in decreasing (from red to pink) importance as determined by orthonormal decomposition (see Fig. 4).

In both PC1 and PC2, the most significant nodes (where there is an autocorrelation) are the basal nodes ‘Ursidae’ (1) and ‘Ursinae-Tremarctinae’ (2). In PC1, the nodes ‘*Ursus* +*H. malayanus’* (6) and ‘*U. arctos*-*U. maritimus’* (10; Fig. 3A), and in PC2, the nodes ‘Tremarctinae’ (3) and ‘*U. americanus+U. thibetanus* - *H. malayanus’* (8) also show a phylogenetic signal (Fig. 3B).

In both PCs, the statistics from R2Max are nonsignificant (*p* = 0.37 for PC1 and *p* = 0.28 for PC2), but significant values were obtained for SkR2k, DMax and SCE (Figs. S6, S8).

In both PCs, a ‘diffuse phylogenetic dependence’, as defined by Ollier, Couteron & Chessell (2006), is observed to the degree to which the phylogenetic history has shaped the evolution of phenotypic characters or life traits. This is given by the presence of a significant departure from H0 in three test statistics (SkR2k, DMax and SCE, while the cumulative orthogram has several values outside the confidence limits Figs. 2D–2F, 2J–2L) and R2Max statistics, which is nonsignificant (see above; Figs. 2C, 2I). The values of the orthogram—thus the portions of interspecific variance—decrease regularly as a function of the complexity value, np, of the nodes. In PC1 and PC2, the variation of the trait is accumulated mostly at the root of the tree, while in the tips of the tree, the variation decreases. According to the cumulative decomposition plots, in all cases, several nodes show values extending beyond the confidence limits built by the Monte Carlo permutations (Fig. 2).

### Abouheif C mean

**Figure 4:**
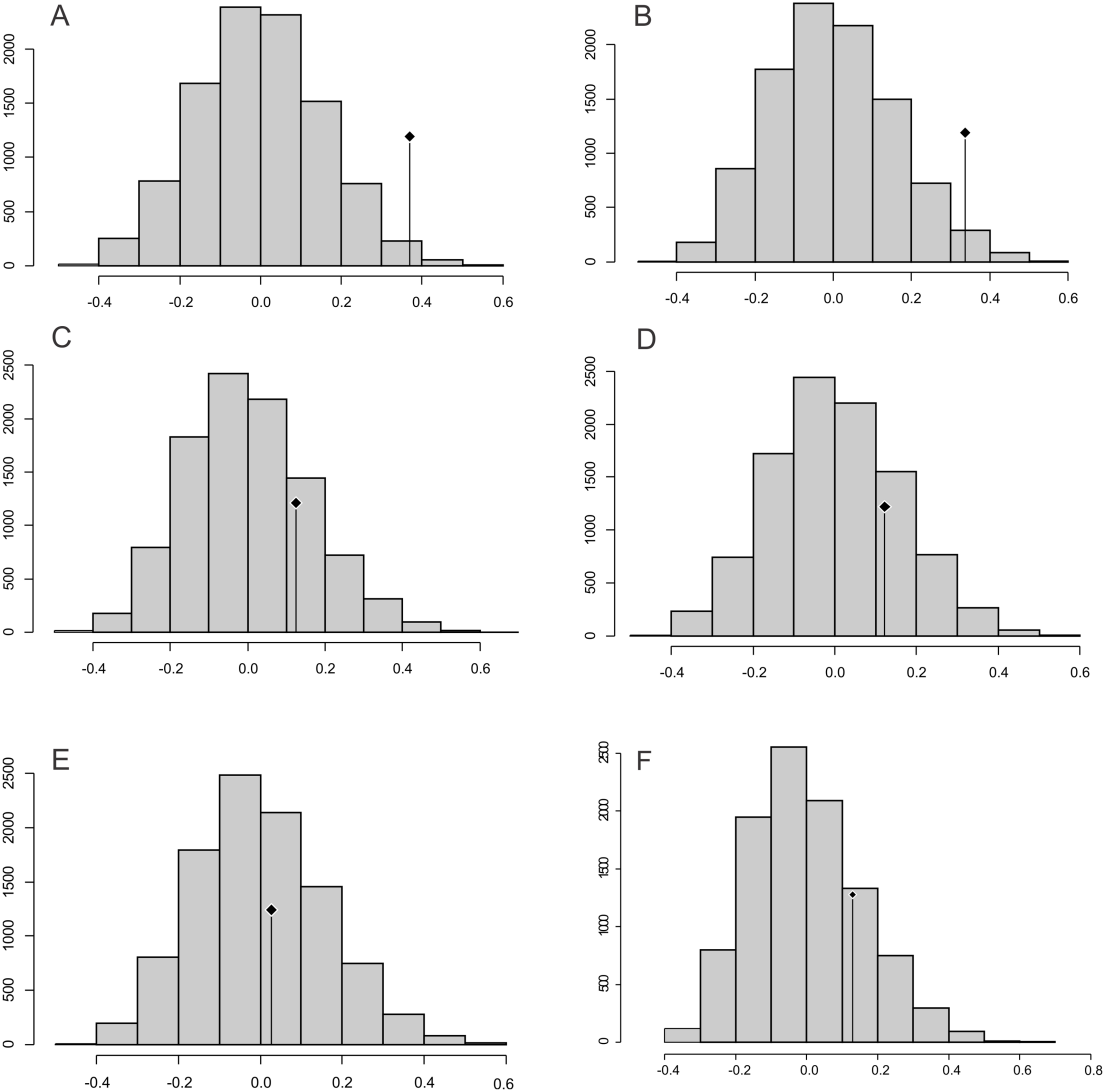
Abouheif C-mean results for the first’s five PC (A–E, respectively) axes and the centroid size (F). Black dots indicate the position of the observed C-mean statistic relative to the H_0_ hypothesis developed by randomizations along the tips of the phylogeny.

The observed position of the C mean statistic is significantly different from the expected sampling distribution of the null hypothesis developed by randomising the tips at a 0.05 alpha for PC1 and PC2; therefore, there is a statistically significant autocorrelation (Figs. 4A, 4B). For PC3, PC4, PC5 and size (CS), the observed position of the C mean is not significantly different than the expected sampling distribution of the null hypothesis developed by randomising the tips at a 0.05 alpha. Therefore, phylogeny is not a significant factor for centroid size; but for shape, closely related taxa are more similar than expected (Figs. 4C–4F).

### Landmark optimisation

**Figure 5:**
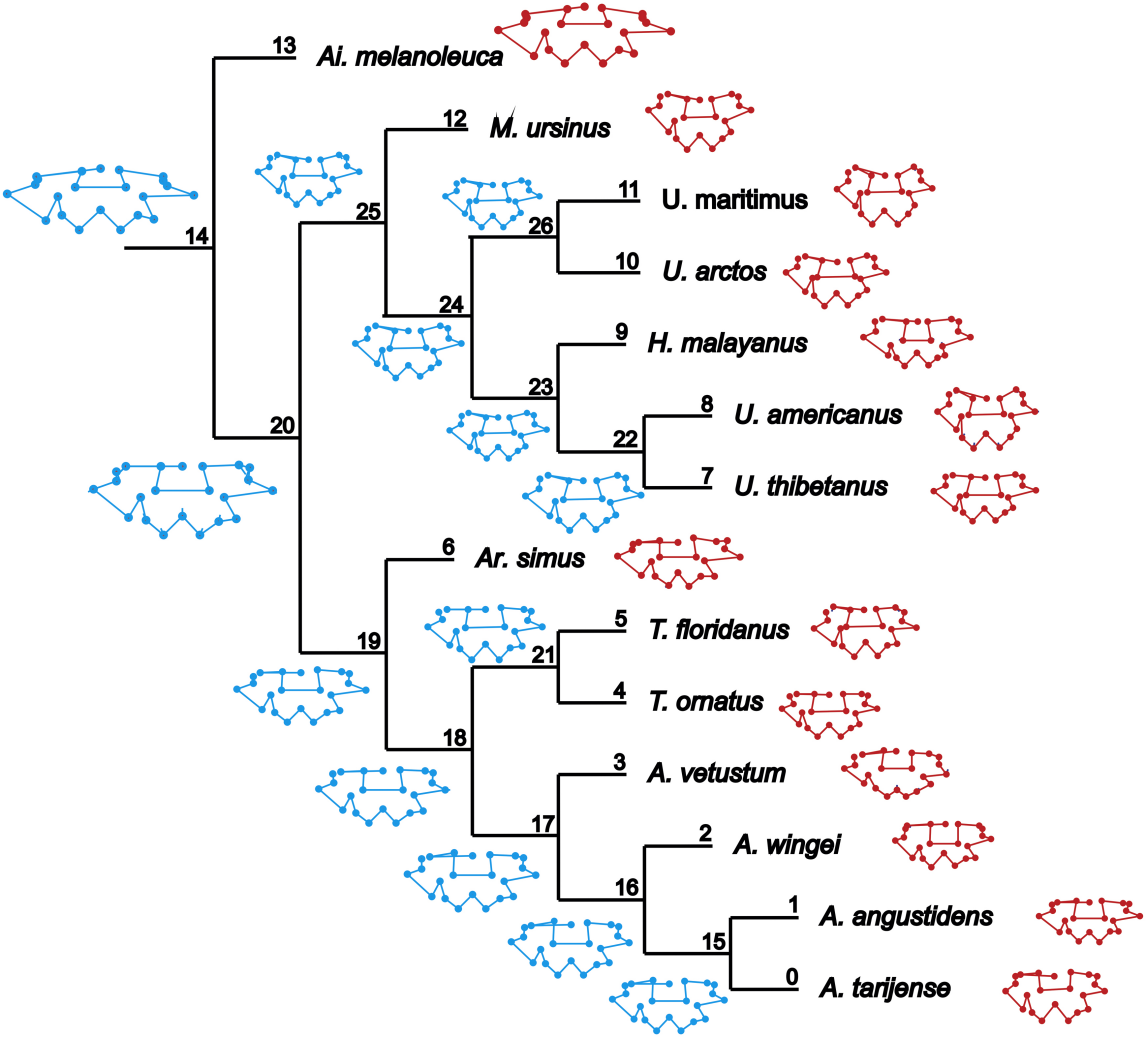
Landmark optimization. Reconstruction of the shape of the basicranium for nodes (in blue) and observed landmark configuration of terminals (in red).

The ancestral configuration of the basicranium was analysed in the nodes that showed a significant phylogenetic signal. The basicranium at the basal node ‘Ursidae’ (Fig. 5, node 14) was reconstructed as antero-posteriorly short and laterally expanded; the basioccipital–basisphenoid contact was anteriorly convex; the otic region was also reduced, while the mastoid processes were wide; the occipital condyles are more anteriorly located and aligned with the paroccipital processes. This morphology coincides with that of *Ailuropoda melanoleuca.* In the node ‘Tremarctinae+Ursinae’ (Fig. 5, node 20), the basicranium is more antero-posteriorly expanded; with a straight basioccipital–basisphenoid contact, the otic region is more expanded but with narrower mastoid processes and occipital condyles posteriorly placed with respect to the paraoccipital processes. In the node ‘Tremarctinae’ (Fig. 5, node 19), the basicranium configuration differs from that of the most inclusive nodes in that it is laterally narrower and antero-posteriorly elongated, differing from that of the node ‘Ursinae’ (Fig. 5, node 25) in having a straight basioccipital–basisphenoid contact and an otic region that is more anteriorly placed and slightly expanded but with wider mastoid processes. In the node ‘Ursinae’, the basicranium is more antero-posteriorly elongated than in more inclusive nodes, and in the node ‘Tremarctinae’, the basioccipital–basisphenoid contact is anteriorly concave, the mastoid processes are narrower, the occipital condyles are posteriorly located, and the otic region is more expanded with a larger tympanic bone.

### Skull proportions

**Figure 6:**
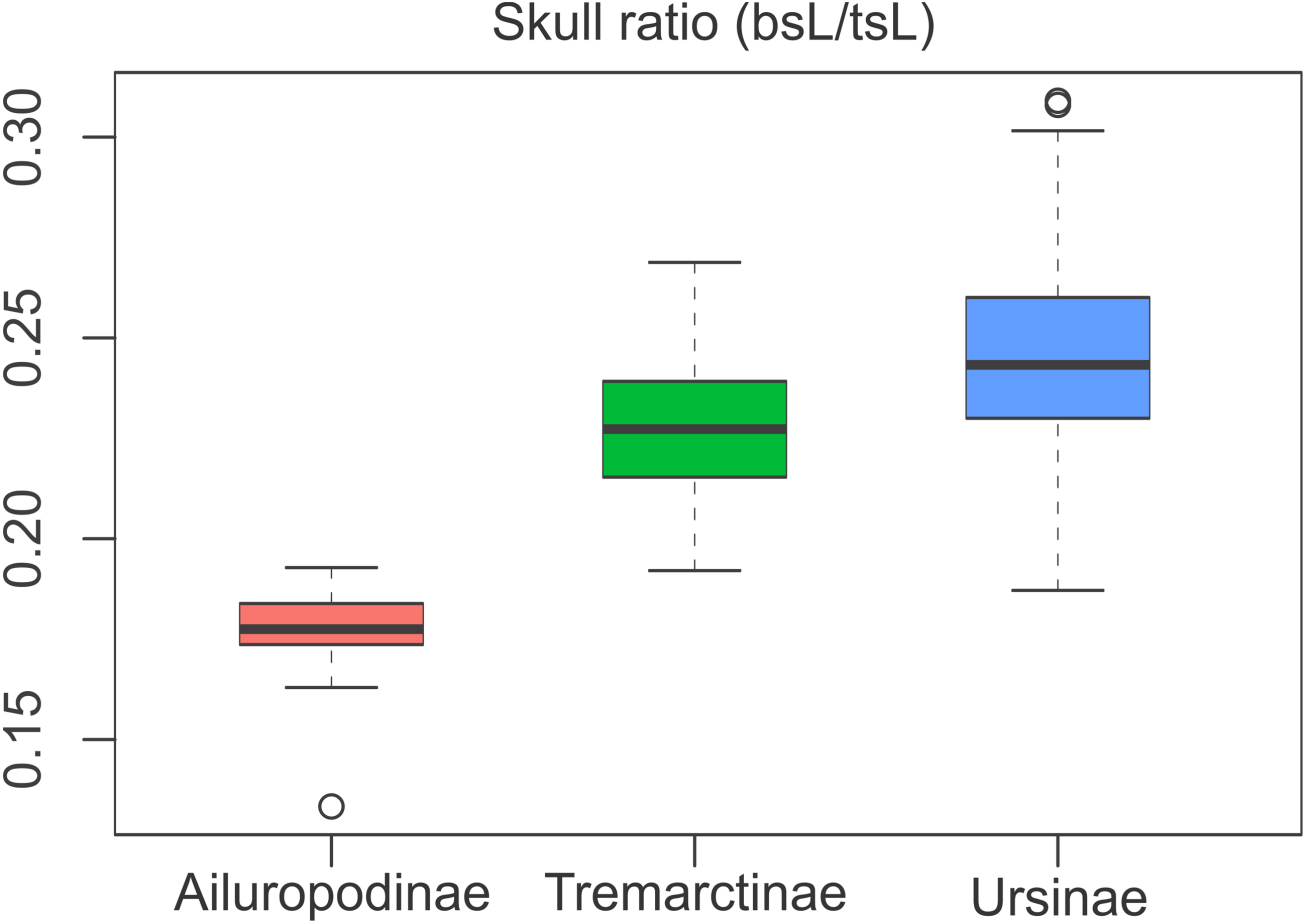
Boxplot of the skull ratio (bsL/tsL ) per subfamilies where significant differences amongst them are observed. Boxes’s floor and roof denote first and third quartile, respectively. Whiskers show 1.5 interquartile range, and blank circles are outliers.

The relative proportions of the basicranium rendered significant differences between subfamilies (Table S3), indicating that the basicranium is comparatively shorter in Ailuropodinae when compared with Tremarctinae and Ursinae and in turn shorter in Tremarctinae than in Ursinae (Fig. 6). Also, a small difference is present in the basicranium within Tremarctinae (i.e., *A. angustidens* and *Ar. simus* presents a skull ratio of 0.20, while *A. wingei* has a skull ratio of 0.26).

## Discussion

According to the morphological variability of the basicranium in ursids, Arnaudo & Fernandez Blanco (2016) found that the distribution of taxa in the morphospace results in groups that are fixed with the recognised clades Tremarctinae, Ursinae and Ailuropodinae and that the Ursinae presents a higher disparity at the basicranium than Tremarctinae (Fig. S16). Among ursids, *Ailuropoda melanoleuca* shows a very distinct (which potentially could be plesiomorphic, although this hypothesis must be tested with future inclusion of additional outgroups; Fig. 5) configuration of the basicranium: rectangular, antero-posteriorly shorter and a wider basicranium, with wide processus mastoideus, short occipital condyles antero-posteriorly located, a ventral border of the foramen magnum anteriorly located, and basioccipital–basisphenoid contact anteriorly convex. A more derived morphology could be found in Ursinae (Fig. 5) with a rhomboidal, more antero-posteriorly elongated and narrower basicranium, with the basioccipital–basisphenoid contact anteriorly concave, otic region more expanded with larger tympanic bone, occipital condyles wider and posteriorly located, ventral margin of the foramen magnum also posteriorly located and foramen postglenoideum anteriorly located. In the current study, a high phylogenetic signal was also obtained in these principal nodes, showing that some ecological and biogeographic factors could be involved in the macroevolution of the braincase shape of ursids. However, this is not reflected in the size because ursids with similar basicranium shapes show different sizes and eating behaviours.

We observed that the node ‘Tremarctinae+Ursinae’ included some major lineages (e.g., *Ursus arctos* and *U. maritimus*) that inhabit open habitats (e.g., grasslands, savannas) and others in closed habitats (e.g., different types of forests; see Table S4). Also, those that live in closed habitats (e.g., black bears, *Helarctos malayanus*) are omnivore-hypocarnivore (feeding mainly on plant matter but incorporating insects and occasionally small mammals), small sized and live in tropical, subtropical or temperate climates (i.e., they do not rely on fat storage for winter). On the other hand, those bears that inhabit open habitats (i.e., *Ursus arctos*, *U. maritimus*) are omnivore–carnivore animals and sometimes feed on plants too, are larger in size and live (or lived in the case of extinct taxa, i.e., *Ar simus* and *A. angustidens*) under much more severe climates, so they present a larger motivation to gain fat during favourable seasons (see Table S4).

**Figure 7:**
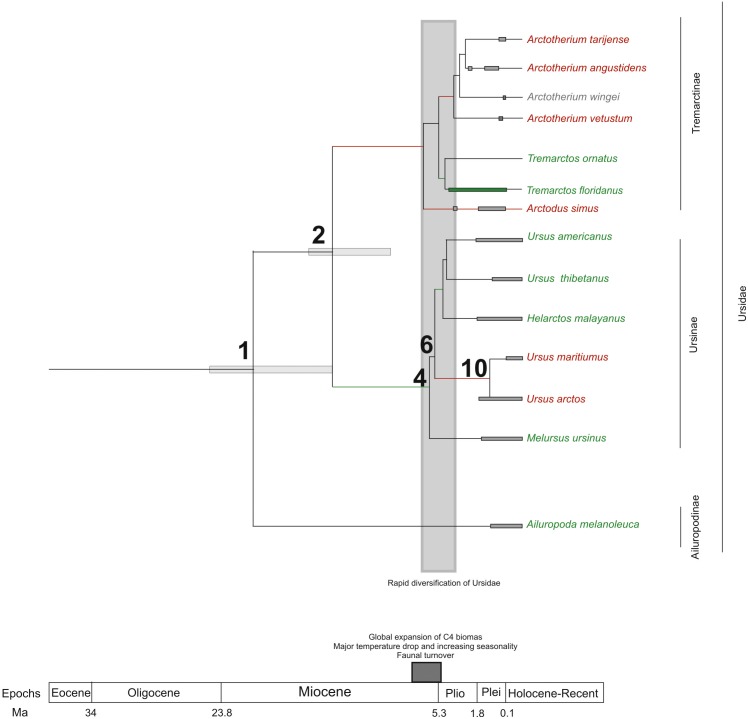
Cladogram in which habitat preferences where scored and optimized. Open habitat are in red lines, while closed habitats are in green lines (Modified from Krause et al., 2008).

If we score and optimise open (red lines) versus closed (green lines) habitat preferences on the cladogram (Fig. 7), it can be seen that at the cladogenetic event that occurred on node 2 (when the Tremarctinae and Ursinae subfamilies differentiated), the preference for open habitats (Tremarctinae) or closed habitats (Ursinae) could have been a factor. These two clades may represent different solutions for balancing energy expenditure, intake, foraging time, fat accumulation and fitness, depending on food availability, foraging efficiency, body size and condition, as suggested by Welch et al. (1997) regarding the frugivory by bears. It is possible that the phylogenetic signal observed in the basicranium shape on node 2 would be related to an early differentiation of these two different evolutionary trends.

Compared with Ursinae, Tremarctinae shows a distinctive basicranium morphology characterised with a straight basioccipital–basiesphenoid contact; the basioccipital area is more expanded in relation to the otic region, which is located anteriorly with a wider mastoid process (PC2 also shows a high phylogenetic signal; Fig. 2). This configuration of the basicranium is practically conservative among Tremarctinae, independent of size variation (∼100 kg to ∼1,200 kg) and diet. Different proportions of the basicranium observed within Tremarctinae respective to the total length of the skull could be related to its diet (Fig. 6). Although all species exhibit almost the same basicranium configuration, the species that consumed higher amounts of animal items (e.g., scavengers such as *A. angustidens* and *Ar. simus*) present a smaller skull ratio and species with high amounts of herbivore items (e.g., *T. floridanus* and *A. wingei*) present the higher skull ratios; however, those species (e.g., *A. vetustum* and *A. tarijense*) with an intermediate proportion of vegetal items in their diets showed intermediate values of skull ratios (Table S5). In this way, the high phylogenetical signal for the node Tremarctinae probably indicates that the general configuration of the basicranium in these bears was obtained early in their evolutionary history (which is in line with (Krause et al., 2008), who stated that Tremarctinae diverged about 12.4 to 15.6 Ma; Fig. 7).

The modifications in the skull of Ursidae could be the result of major climatic changes that occurred during the early cladogenesis of the largest bear clades; Krause et al. (2008), see their Figs. 1 and 2) observed an explosive radiation of Ursidae at or just after the Mio-Pliocene boundary, suggesting that it was related to the paleoecological context of the Mio-Pliocene boundary, which had the following factors: (1) global increase in C4 biomass, where open wooded grassland habitats replaced the earlier, less seasonal woodland forest, resulting in habitat diversity reduction (Ehleringer et al., 1991; Ehleringer, Cerling & Helliker, 1997); (2) the Late Miocene carbon shift that resulted in a latitudinal gradient of C3/C4 grasses, with C3 grasses predominating in colder, more polar regions and C4 grasses predominating in temperate and tropical regions (MacFadden, 2000); (3) C4 biomass expanded in tropical to temperate regions; (4) major temperature drops came with an increase of seasonality; (5) terrestrial environments on all continents (except Antarctica) underwent major changes in fauna at the Mio-Pliocene boundary (60–80 genera of mammals were removed both in North America and Eurasia; Savage & Russell, 1983; Web, 1983; Webb, 1984; Cerling, Ehleringer & Harris, 1998; (6) the Plio-Pleistocene predator guild differed from all previous guilds in that it included a variety of carnivores with clear long-distance pursuit abilities. In this regard, massive predators were replaced by omnivorous bears and more specialised carnivores, such as felids and hyaenids (Van Valkenburgh, 1999).

These changes in habitat and food sources affected bears’ ecology during the differentiation of the main clades. They became adaptable opportunists (e.g., Peyton, 1980; Raine & Kansas, 1990; Wong, Servheen & Ambu, 2002; Hansen et al., 2010; Donohue, 2013), and dietary versatility may have allowed ursids to persist during the dramatic habitat fluctuations of the Pleistocene and Holocene, favouring the wider distribution of the members of this family (Krause et al., 2008).

## Concluding Remarks

Our results indicate that the variation of the basicranium shape (but not size) is significantly correlated with the topology of the cladogram, which depicts phylogenetic relationships. That is, the basicranium shape appears to be explained by a common heritage. The most significant nodes where phylogenetic autocorrelation of the basicranium shape was detected included basal differentiation of the major ursid lineages, indicating that cladogenesis of pandas, Ursinae and Tremarctinae was caused by evolutionary trends, and the resulting similarity among taxa analysed here could be explained by long-lasting influential factors. In this sense, early differentiation of Tremarctinae and Ursinae could be related to historical events involving different environmental food preferences, which could be the factors that influenced the subsequent evolution of basicranium shape.

Our results of the basicranium/skull length ratios indicate that in Tremarctinae basicranium, size was not determined largely by phylogeny but rather by other factors such as adaptive responses to climatic changes and competition with other carnivores.

##  Supplemental Information

10.7717/peerj.6597/supp-1 Supplemental Information 1 Summary of the results of Arnaudo and Fernadez Blanco 2016

10.7717/peerj.6597/supp-2 Supplemental Information 2 Supplementary material

10.7717/peerj.6597/supp-3 Supplemental Information 3 Raw data Basicranium

10.7717/peerj.6597/supp-4 Table S1 List of the specimens used in the geometric morfometric analysis Abbreviations: F, female; M, male; NS, not sexed.

10.7717/peerj.6597/supp-5 Table S2 Pc scores from (Arnaudo & Fernandez Blanco, 2016)

10.7717/peerj.6597/supp-6 Table S3 Non-parametrical test for differences in skull ratio between subfamilies in this study

10.7717/peerj.6597/supp-7 Table S4 Summary of the habitat, diet, weight and hibernation/dormancy of Ursinae bears

10.7717/peerj.6597/supp-8 Table S5 Average of the skull ratio of the Tremarctinae species included in this study

## Additional Information and Declarations

### Data Availability

The following information was supplied regarding data availability: The raw data are available in the Supplemental Files.
