# Supplementary material for: Phylogenetic signal analysis in the basicranium of Ursidae (Carnivora, Mammalia)
Source: PeerJ. 2019 Mar 15;7:e6597. doi: 10.7717/peerj.6597 (PMC6422017; doi:10.7717/peerj.6597)
Supplement: Supplemental Information 1 [file peerj-07-6597-s001.docx]

**Summary of Arnaudo and Fernádez Blanco, 2016.**

**Detailed material and method of Arnaudo & Fernandez Blanco (2016)**

The variation of the basicranium of Ursidae was analyzed using 2D geometric morphometric. The authors studied the basicranium of adult specimens of ursids of the three subfamilies (Table S1, Supplementary material). Twenty two type I and II landmarks were established (Table S2, Supplementary material). Shape variation was analyzed using a principal component analysis and a regression of the landmark coordinates against the logarithm of centroid size. Since CS has proposed to behave in a similar way to the body size (Hood, 2000), herein it will be considered as a body size proxy.


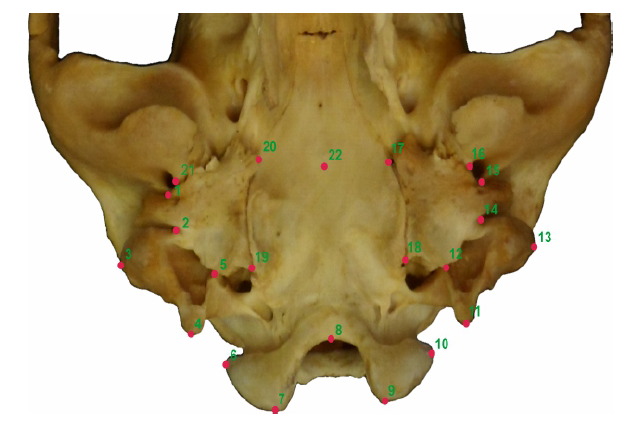


Landmark visualization used in the geometric morphometric analysis, on the *Tremarctos ornatus basicranium*.

Table for detailed definition of the 2D Landmarks used in the geometric morphometric analysis (see figure S1 in Supplemental material for the graphic location)

| **No** | **Definition** | **LM type** |
| --- | --- | --- |
| **1 and 15** | **Most lateral point of the squamosal-tympanic suture.** | **Type I** |
| **2 and 14** | **Most lateral contact between the tympanic and the mastoid process.** | **Type I** |
| **3 and 13** | **Most external point of the mastoid process.** | **Type II** |
| **4 and 11** | **Posterior point of the paraoccipital process.** | **Type II** |
| **5 and 12** | **Most lateral point of the paraoccipital process- tympanic suture.** | **Type I** |
| **6 and 10** | **Lateral point of the condyle.** | **Type II** |
| **7 and 9** | **Posterior point of the condyle.** | **Type II** |
| **8** | **Medial point of the foramen magnum.** | **Type II** |
| **16 and 21** | **Medial edge of the postglenoid foramen.** | **Type II** |
| **17 and 20** | **Contact between tympanic, basioccipital and basiesfenoid** | **Type I** |
| **18 and 19** | **Poterior contact between the tympanic and basioccipital.** | **Type I** |
| **22** | **Contact between basioccipital and basiesfenoid in the middle line.** | **Type II** |

**Results**

Along PC1 (Fig. S16), it was observed that there was a clear separation of the sample into two groups. One of the groups (Ursinae+Tremarctinae) is located in the range -0,15 to 0,10, on the left area of the morphospace, while the other group (*Ailuropoda*) occupies the right area of the morphospace, and is in a range of values from 0,10 to 0,22 approximately. *Ailuropoda* is the only species which occupies a clear separated position in the morphospace on PC1, with a laterally expanded and posteriorly shorter basicranium. Regarding the other assemblage (Tremarctinae+Ursinae), even if it is observed with a certain overlap of specimens, there is a clear difference in the morphological space occupied by ursines (range -0.15 to ~0.5) and tremarctines (range 0 to -0.1). This group is characterized for longer and narrower basicranium (e.g., *U. maritimus*; PC1), but antero-laterally expanded, with the posterior area anteriorly and laterally compressed at the upper region of the morphospace (e.g, *H. malayanus*).

Ursidae species in the morphospace (PC1- PC2 planes) shows two major groups, diverging along the PC1 axis (Fig. S16); individuals located towards negative values presents the basicranium more antero-posteriorly elongated and narrower. The anterior region of the basicranium presents a “U” shape (Fig S16). Towards the positive values, the specimens present a basicranium anteroposteriorly shorter and wider. The anterior region of the basicranium is almost straight. (Fig S16).

Along PC2, both in the negative and positive values, the basicranium presents a similar rhomboidal shape, however some differences were found: toward negative values the occipital condyles are more laterally located; the anterior border of the foramen magnum is placed anteriorly; the mastoid process are postero-lateral; and the paraoccipital process are wider than the consensus and the anterior region of the basicranium is narrower (Fig S16). Towards the positive values, the basicranium show narrower occipital condyles, as well as the paraoccipital process; the anterior border of the foramen magnum is posteriorly located; and the mastoid process are antero-lateral expanded, being wider than the consensus as the anterior region of basicranium (Fig S16).

Within the Ursinae subfamily, it was observed that a certain grouping; *Ursus arctos* is closer to *U. maritimus*, disposed in a vertical strip, and *M. ursinus* is proximal to those species. On the other hand, *U. americanus* specimens are located closely to those of *H. malayanus.* This arrangement was observed not only in the morphospace but also in the dot plots graphs obtained from the Orthonormal decomposition of variance, even for the PC1 and PC2.

In relation to the PC2, Tremarctinae and Ailuropodinae are mainly distributed over negative values while Ursinae specimens are distributed all over the axis (Fig. S16).
