# Supplementary material for: Phylogenetic signal analysis in the basicranium of Ursidae (Carnivora, Mammalia)
Source: PeerJ. 2019 Mar 15;7:e6597. doi: 10.7717/peerj.6597 (PMC6422017; doi:10.7717/peerj.6597)
Supplement: Supplemental Information 2 [file peerj-07-6597-s002.pdf]

Cladogram B

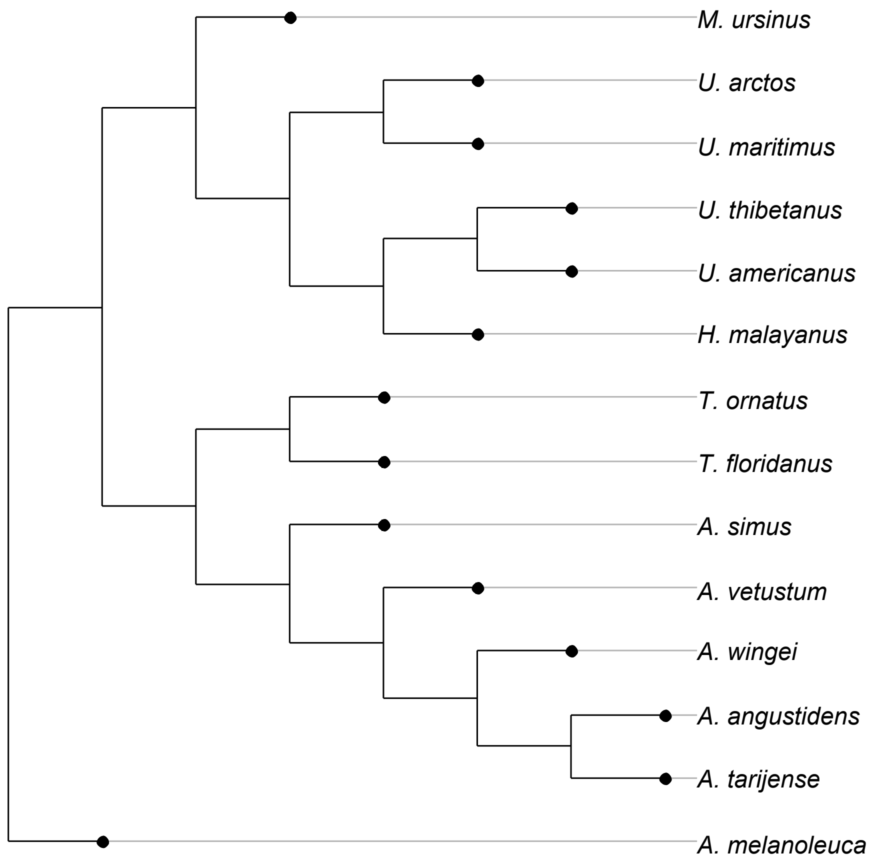

Fig. S1- Phylogenetical hypotesis of Tremarctinae tested. A- Cladogram A: developed by Mitchell et al. (2016), based in molecular characters, takes *Arctodus* as a sister group of the clade formed by *Arctotherium* + *Tremarctos.* B- Cladogram B: based on morphological characters (Soibelzon, 2002), consider the spectacle bear clade (*Tremarctos floridanus* and *T. ornatus*) as a sister group of the short-faced bear clade (this includes the members of *Arctodus* and *Arctotherium*).

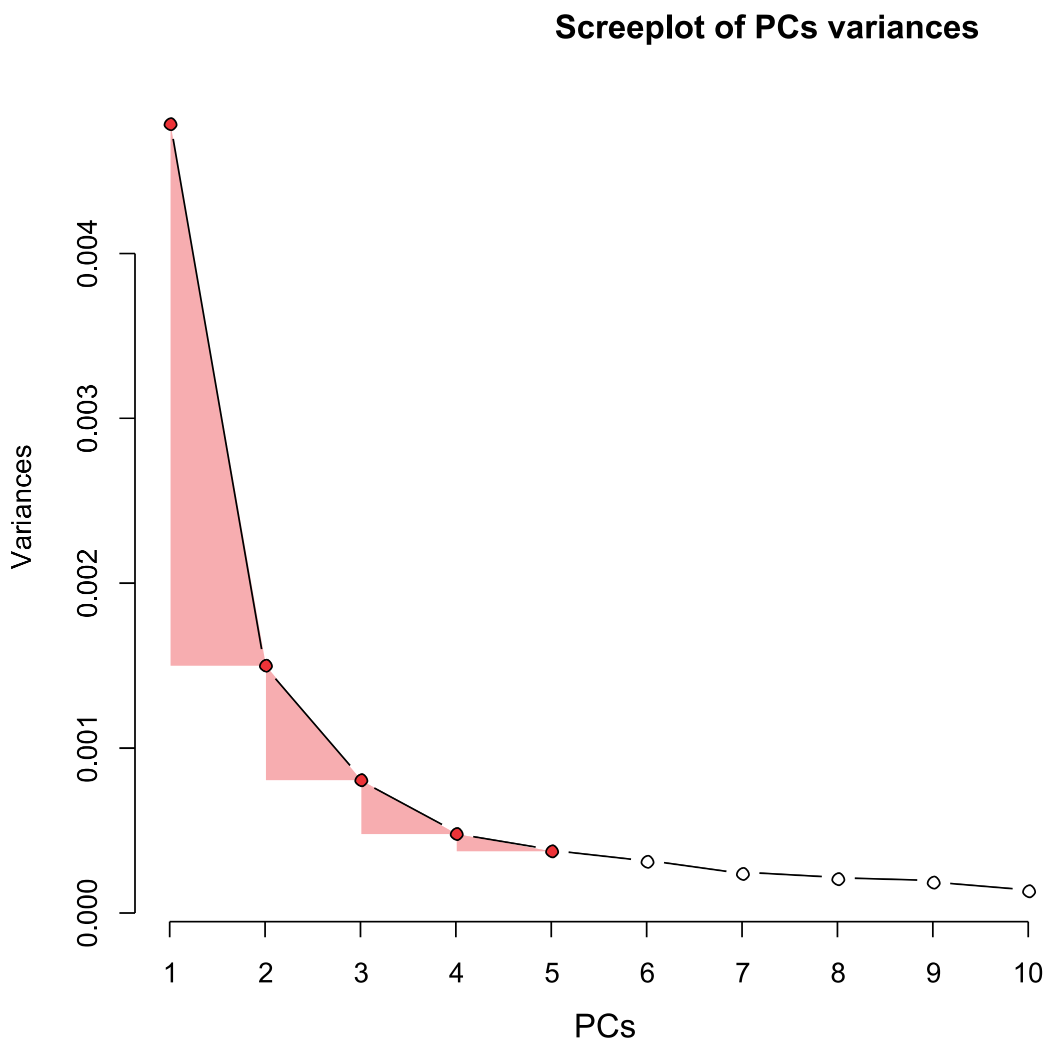

Fig. S2- Scaterplot of the explained variance for the Pcs scores.

Fig.S3- Orthonormal bases for cladogram A (above) and B (below): the observed matrix of orthonormal vectors (orthobases) is depicted, ordered from left to right by decreasing value of explained tree complexity.

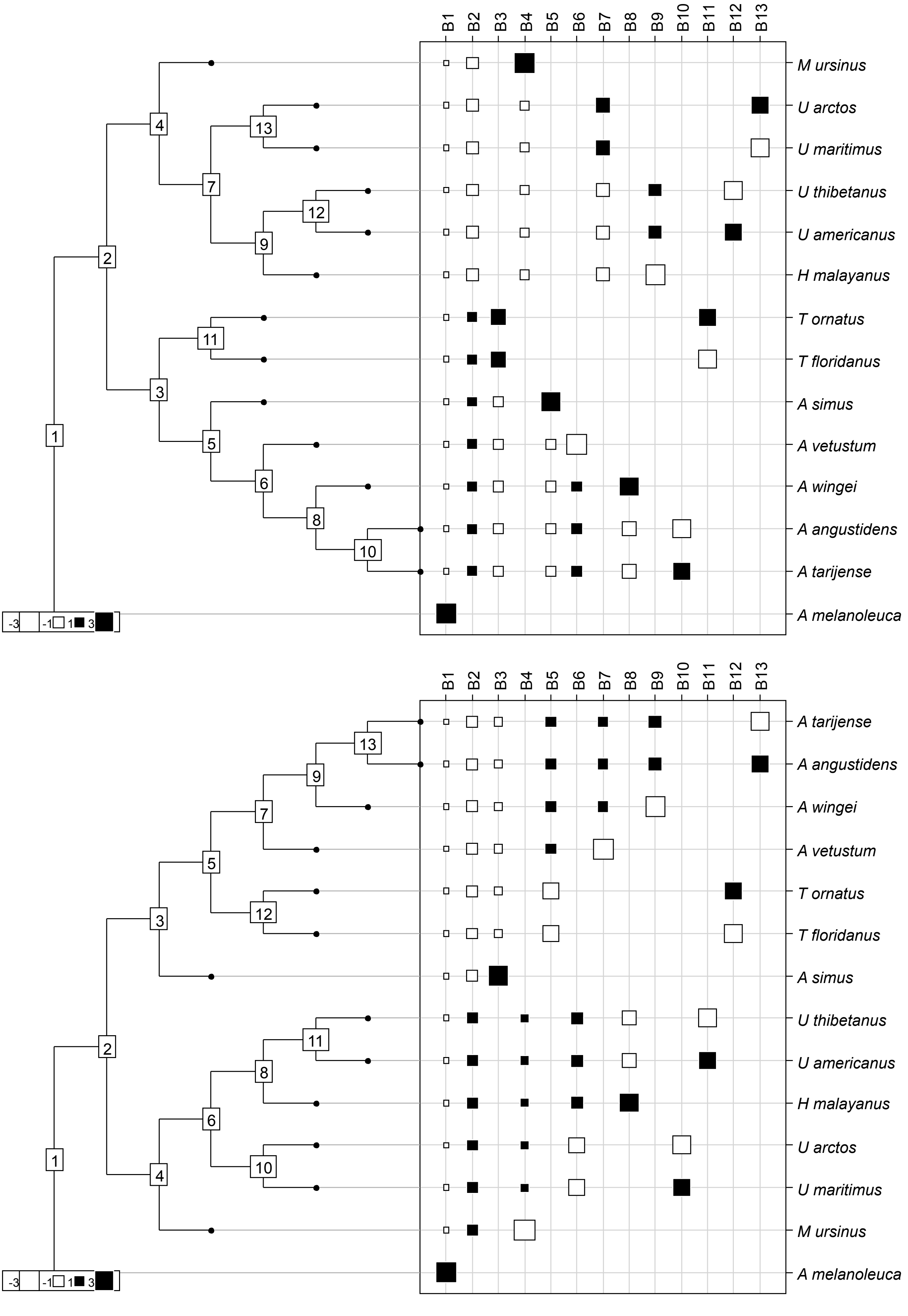

Orthonormal variance decomposition results for cladogram A (CS)

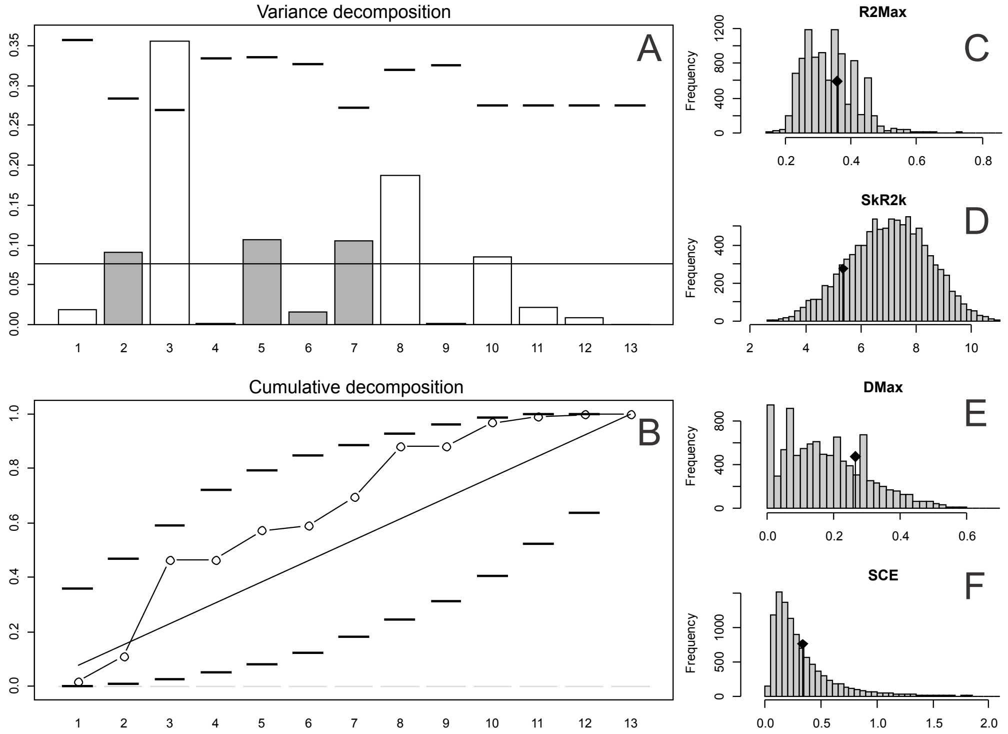

Fig. S4 - Orthonormal decomposition results of CS for Cladogram A. (A) Orthogram plot: height of bars is proportional to the squared coefficients (white and grey bars represents positive and negative coefficients); dashed line is the upper confidence limit at 5 %, built from Monte Carlo permutations; horizontal solid line is the mean value; (B) Cumulative orthogram plot: circles represent observed values of cumulated squared coefficients (vertical axis); the expected values under H0 are disposed on the straight line; dashed lines represent the bilateral confidence interval; (C–F) Histograms of observed values of the four statistic tests: black dot depicts the observed parameter value.

Non-parametric tests for Orthonormal decomposition

	Test	Obs	Std.Obs	Alter	Pvalue
1	R2Max	0.3562202	0.2554424	greater	0.4119
2	SkR2k	5.3593861	-1.1583641	less	0.1349
3	Dmax	0.2665491	0.7059532	two-sided	0.5407
4	SCE	0.3358269	0.1005397	greater	0.3224

Most significant orthobases

3 8 5 7 2 10 11 1 6 12

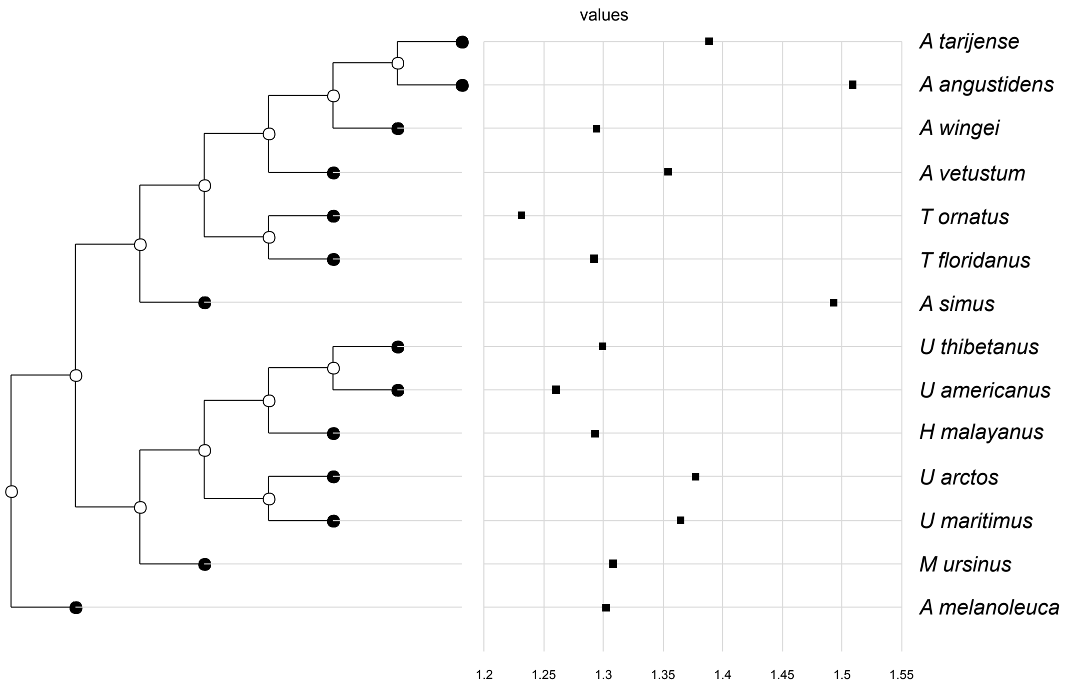

Dotplot of Centroid Size for cladogram A

Orthonormal variance decomposition results for cladogram B (CS)

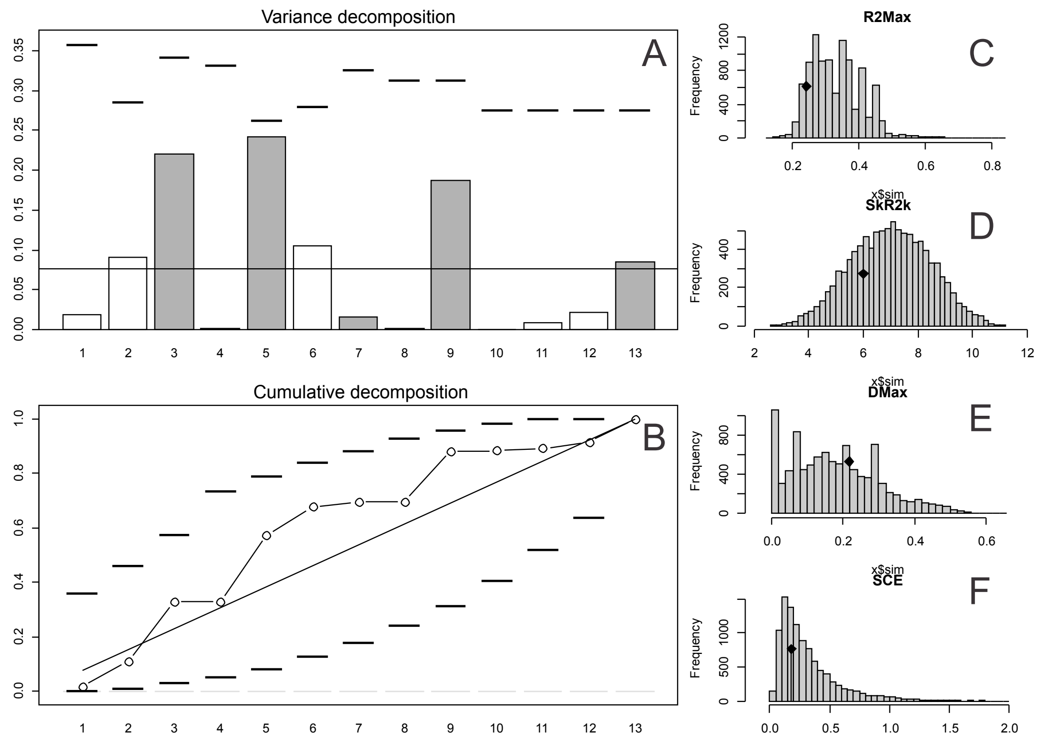

Fig. S5 - Orthonormal decomposition results of CS for Cladogram B. (A) Orthogram plot: height of bars is proportional to the squared coefficients (white and grey bars represents positive and negative coefficients); dashed line is the upper confidence limit at 5 %, built from Monte Carlo permutations; horizontal solid line is the mean value; (B) Cumulative orthogram plot: circles represent observed values of cumulated squared coefficients (vertical axis); the expected values under H0 are disposed on the straight line; dashed lines represent the bilateral confidence interval; (C–F) Histograms of observed values of the four statistic tests: black dot depicts the observed parameter value.

Non-parametric tests for Orthonormal decomposition

	Test	Obs	Std.Obs	Alter	Pvalue
1	R2Max	0.2422607	-1.1895770	greater	0.9034
2	SkR2k	5.9927991	-0.7020069	less	0.2559
3	Dmax	0.2170296	0.3051122	two-sided	0.7756
4	SCE	0.1813408	-0.5411051	greater	0.6415

Most significant orthobases

5 3 9 6 2 13 12 1 7 11

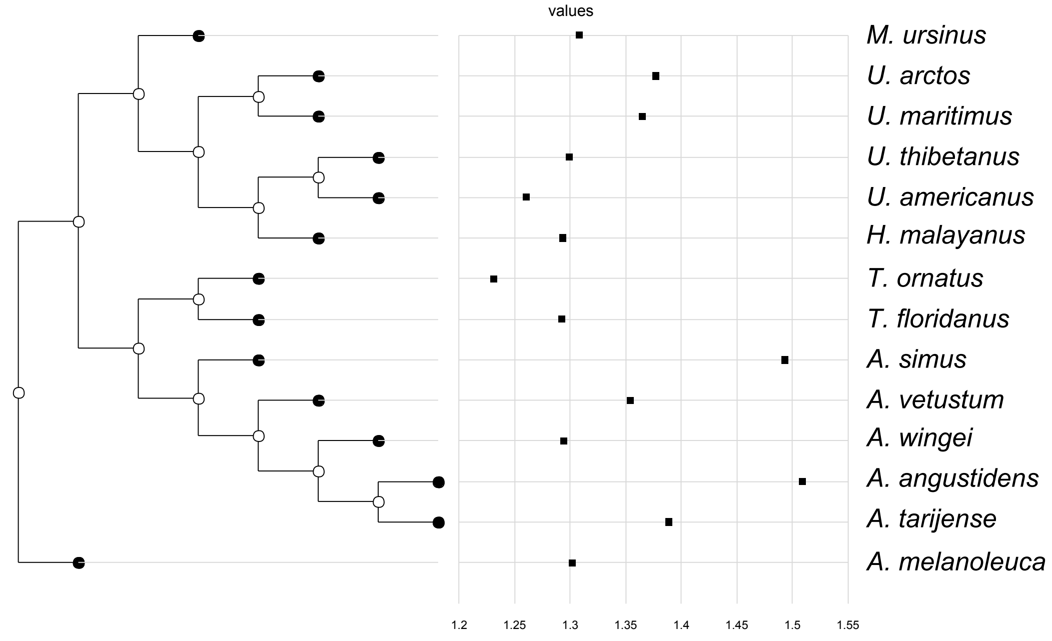

Dotplot of Centroid Size for cladogram B

Orthonormal variance decomposition results for cladogram A (PC1)

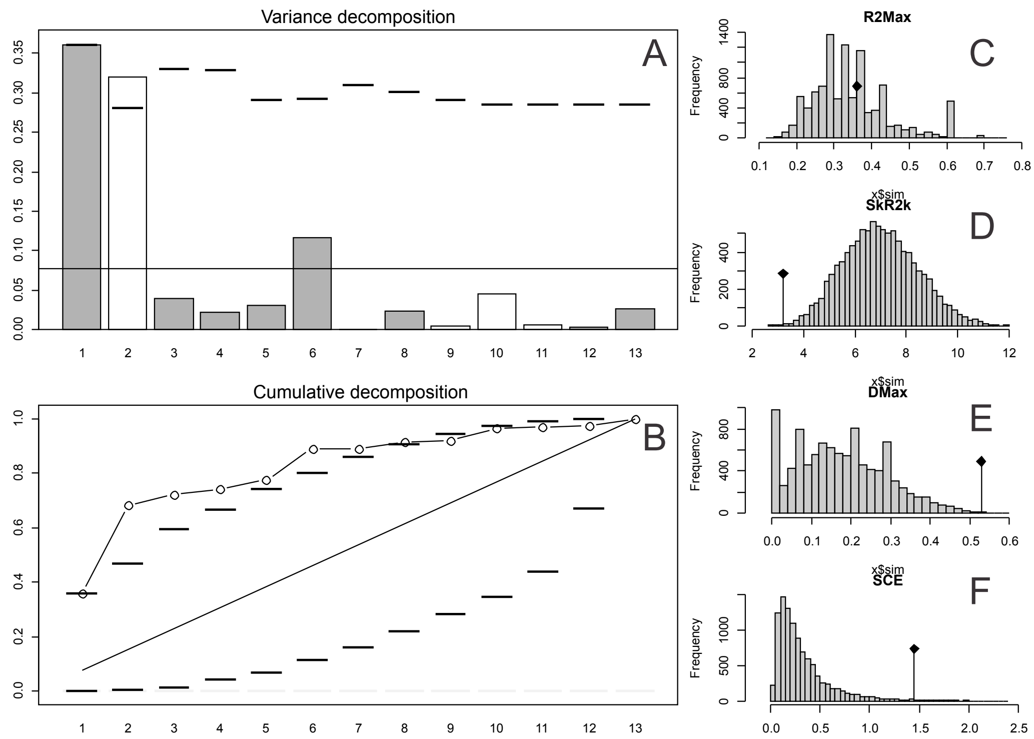

Fig. S6 - Orthonormal decomposition results of PC1 for Cladogram A. (A) Orthogram plot: height of bars is proportional to the squared coefficients (white and grey bars represents positive and negative coefficients); dashed line is the upper confidence limit at 5 %, built from Monte Carlo permutations; horizontal solid line is the mean value; (B) Cumulative orthogram plot: circles represent observed values of cumulated squared coefficients (vertical axis); the expected values under H0 are disposed on the straight line; dashed lines represent the bilateral confidence interval; (C–F) Histograms of observed values of the four statistic tests: black dot depicts the observed parameter value.

Non-parametric tests for Orthonormal decomposition

	Test	Obs	Std.Obs	Alter	Pvalue
1	R2Max	0.3613735	0.171596	greater	0.3745
2	SkR2k	3.1861610	-2.656934	less	0.0006
3	Dmax	0.5280973	3.112760	two-sided	0.0014
4	SCE	1.4529904	4.278821	greater	0.0062

Most significant orthobases

1 2 6 10 3 5 13 8 4 11

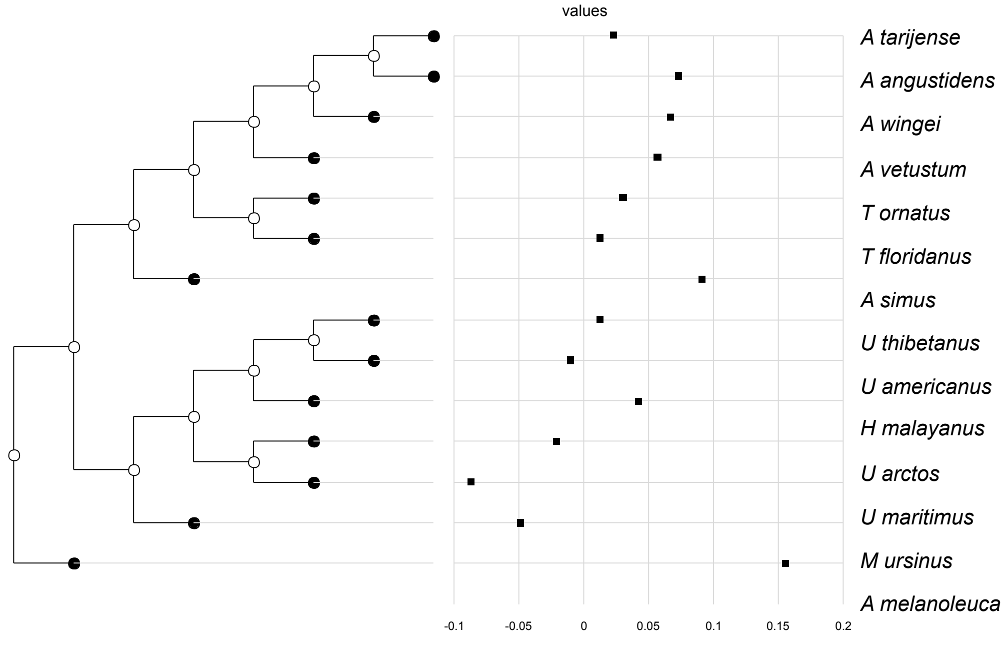

Dotplot of PC1 for cladogram A

Orthonormal variance decomposition results for cladogram B (PC1)

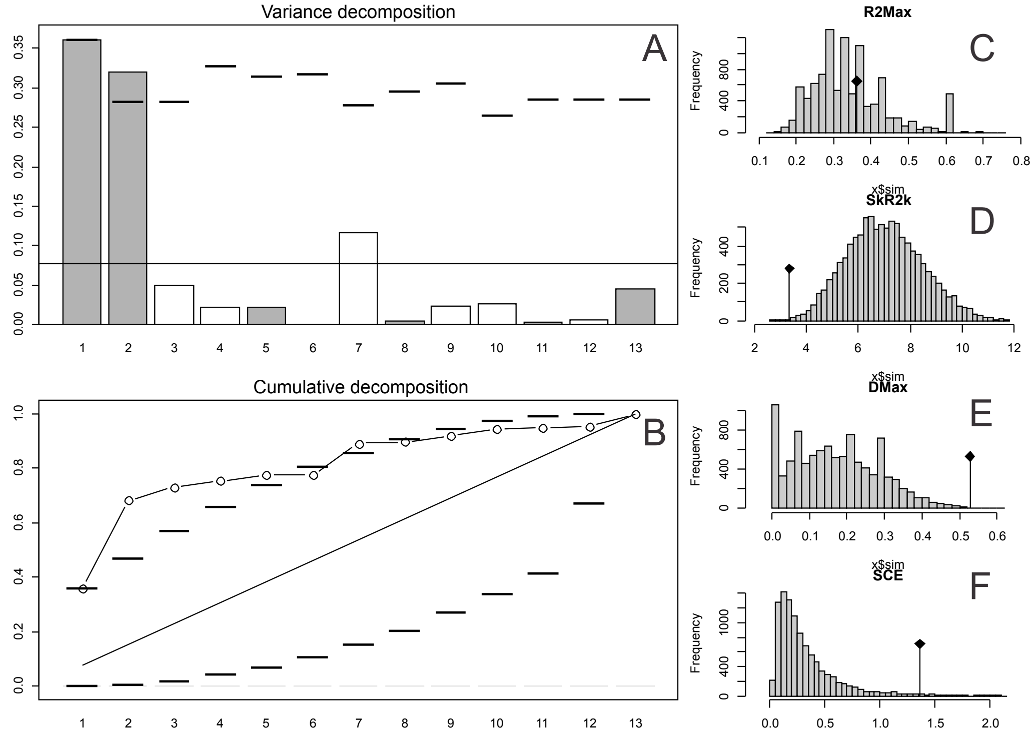

Fig. S7 - Orthonormal decomposition results of PC1 for Cladogram B. (A) Orthogram plot: height of bars is proportional to the squared coefficients (white and grey bars represents positive and negative coefficients); dashed line is the upper confidence limit at 5 %, built from Monte Carlo permutations; horizontal solid line is the mean value; (B) Cumulative orthogram plot: circles represent observed values of cumulated squared coefficients (vertical axis); the expected values under H0 are disposed on the straight line; dashed lines represent the bilateral confidence interval; (C–F) Histograms of observed values of the four statistic tests: black dot depicts the observed parameter value.

Non-parametric tests for Orthonormal decomposition

	Test	Obs	Std.Obs	Alter	Pvalue
1	R2Max	0.3613735	0.1717987	greater	0.3771
2	SkR2k	3.3591427	-2.5397228	less	0.0013
3	Dmax	0.5280973	3.1234022	two-sided	0.0012
4	SCE	1.3610863	3.8365876	greater	0.0103

Most significant orthobases

1 2 7 3 13 10 9 4 5 12

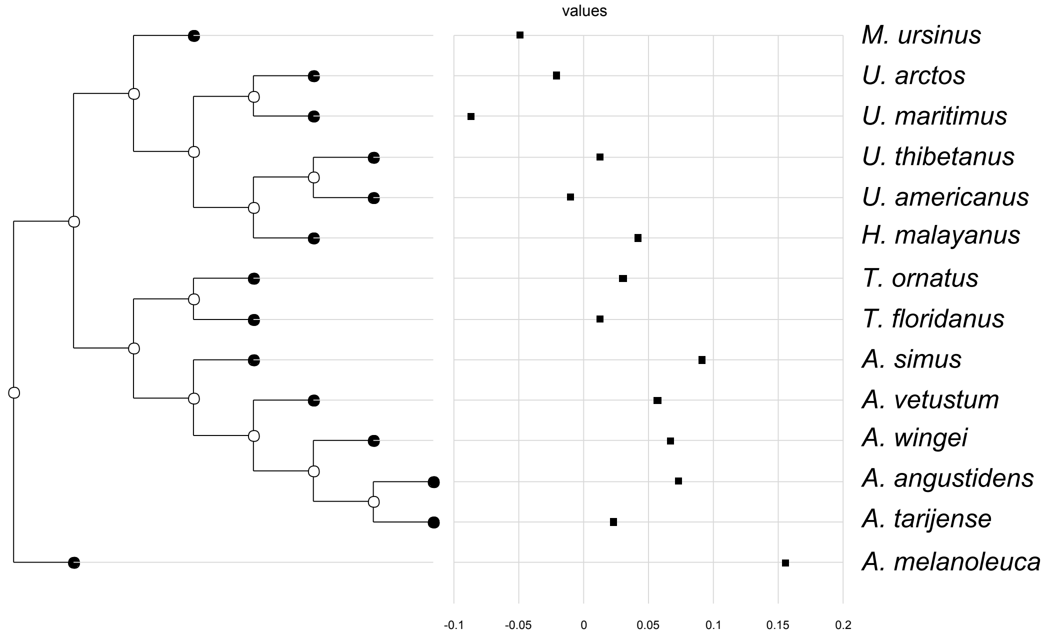

Dotplot of PC1 for cladogram B

Orthonormal variance decomposition results for cladogram A (PC2)

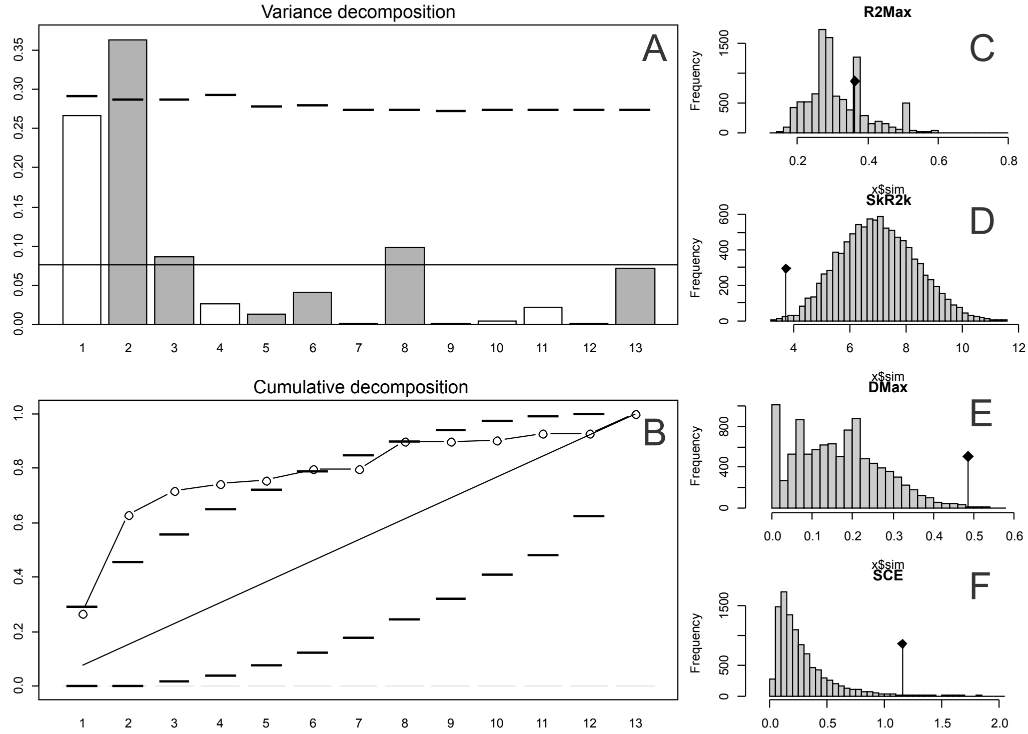

Fig. S8 - Orthonormal decomposition results of PC2 for Cladogram A. (A) Orthogram plot: height of bars is proportional to the squared coefficients (white and grey bars represents positive and negative coefficients); dashed line is the upper confidence limit at 5 %, built from Monte Carlo permutations; horizontal solid line is the mean value; (B) Cumulative orthogram plot: circles represent observed values of cumulated squared coefficients (vertical axis); the expected values under H0 are disposed on the straight line; dashed lines represent the bilateral confidence interval; (C–F) Histograms of observed values of the four statistic tests: black dot depicts the observed parameter value.

Non-parametric tests for Orthonormal decomposition

	Test	Obs	Std.Obs	Alter	Pvalue
1	R2Max	0.3636860	0.5456817	greater	0.2842
2	SkR2k	3.7329691	-2.4344016	less	0.0030
3	Dmax	0.4859883	2.9693713	two-sided	0.0036
4	SCE	1.1554902	3.8431876	greater	0.0095

Most significant orthobases

2 1 8 3 13 6 4 11 5 10

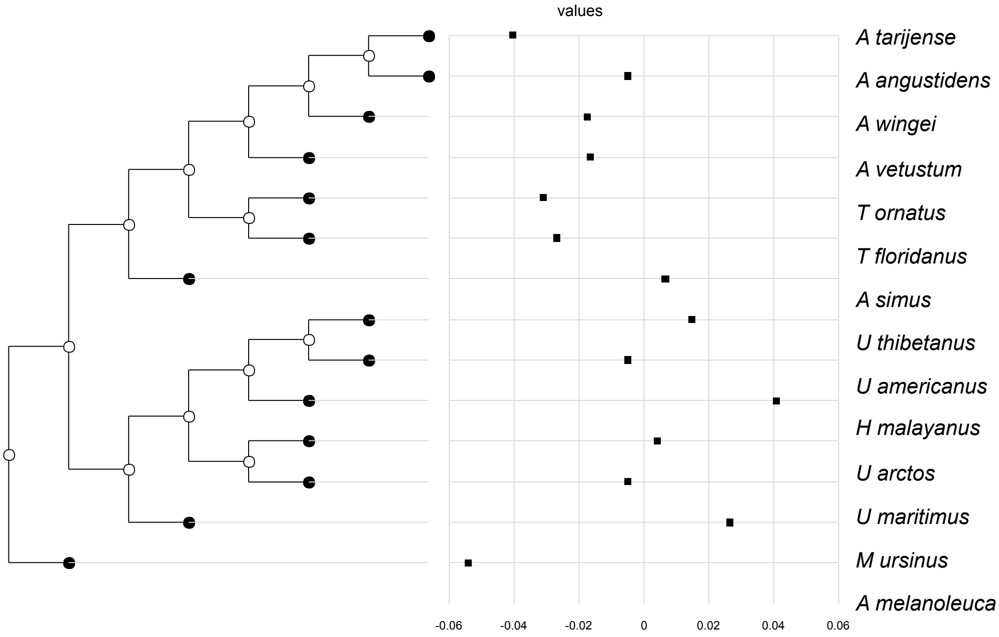

Dotplot of PC2 for cladogram A

Orthonormal variance decomposition results for cladogram B (PC2)

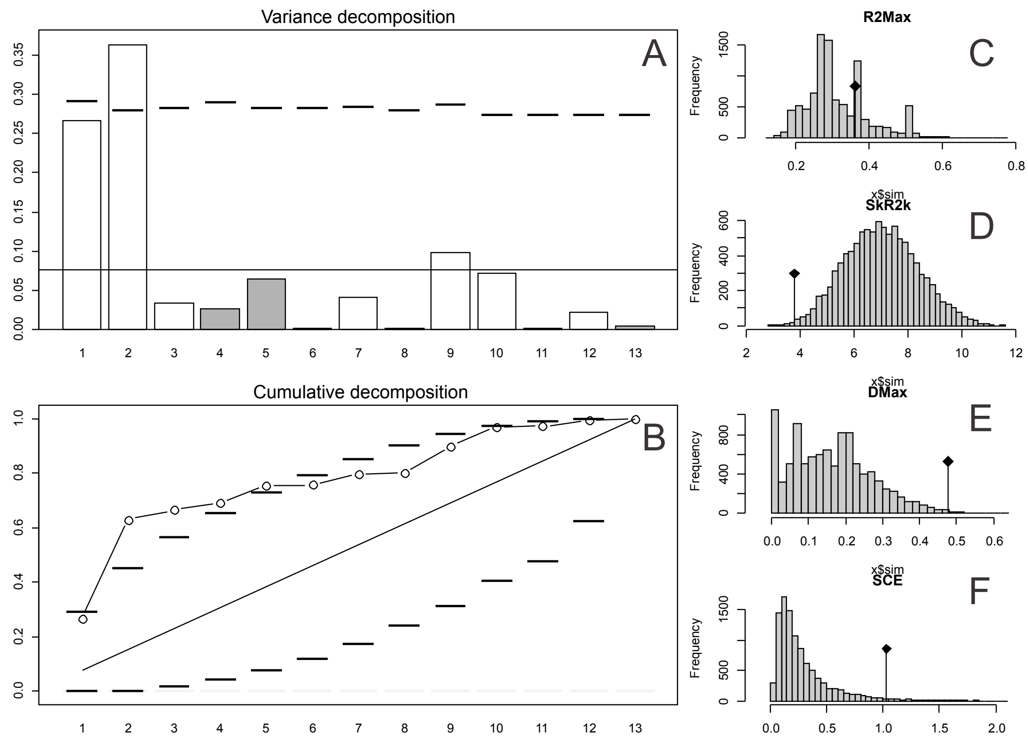

Fig. S9 - Orthonormal decomposition results of PC2 for Cladogram B. (A) Orthogram plot: height of bars is proportional to the squared coefficients (white and grey bars represents positive and negative coefficients); dashed line is the upper confidence limit at 5 %, built from Monte Carlo permutations; horizontal solid line is the mean value; (B) Cumulative orthogram plot: circles represent observed values of cumulated squared coefficients (vertical axis); the expected values under H0 are disposed on the straight line; dashed lines represent the bilateral confidence interval; (C–F) Histograms of observed values of the four statistic tests: black dot depicts the observed parameter value.

Non-parametric tests for Orthonormal decomposition

	Test	Obs	Std.Obs	Alter	Pvalue
1	R2Max	0.363686	0.5223974	greater	0.2911
2	SkR2k	3.791600	-2.4016221	less	0.0035
3	Dmax	0.476947	2.8781098	two-sided	0.0044
4	SCE	1.033076	3.2027479	greater	0.0175

Most significant orthobases

2 1 9 10 5 7 3 4 12 13

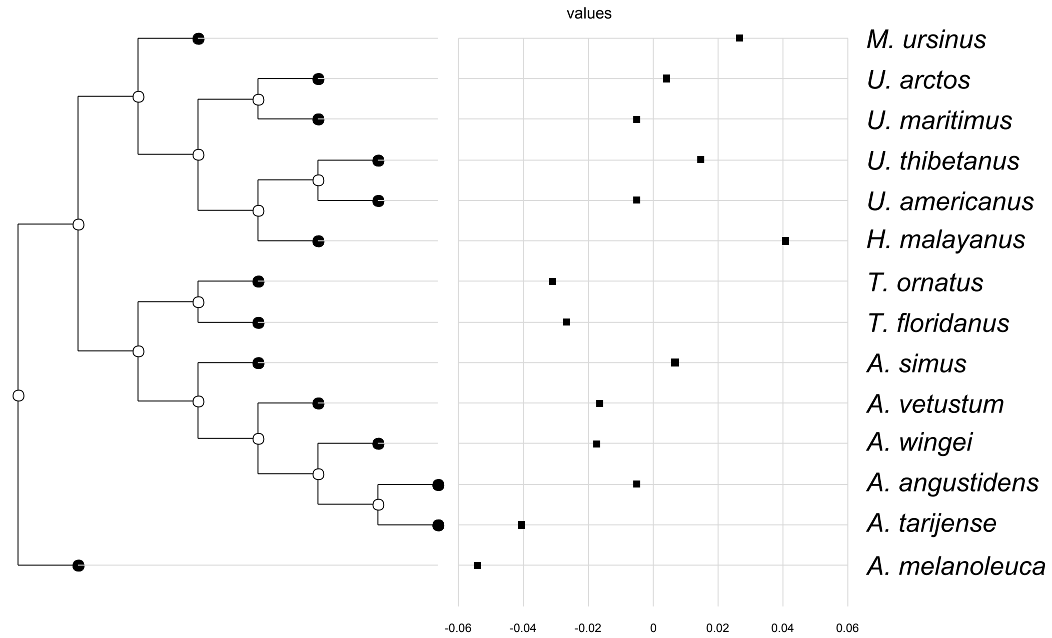

Dotplot of PC2 for cladogram B

Orthonormal variance decomposition results for cladogram A (PC3)

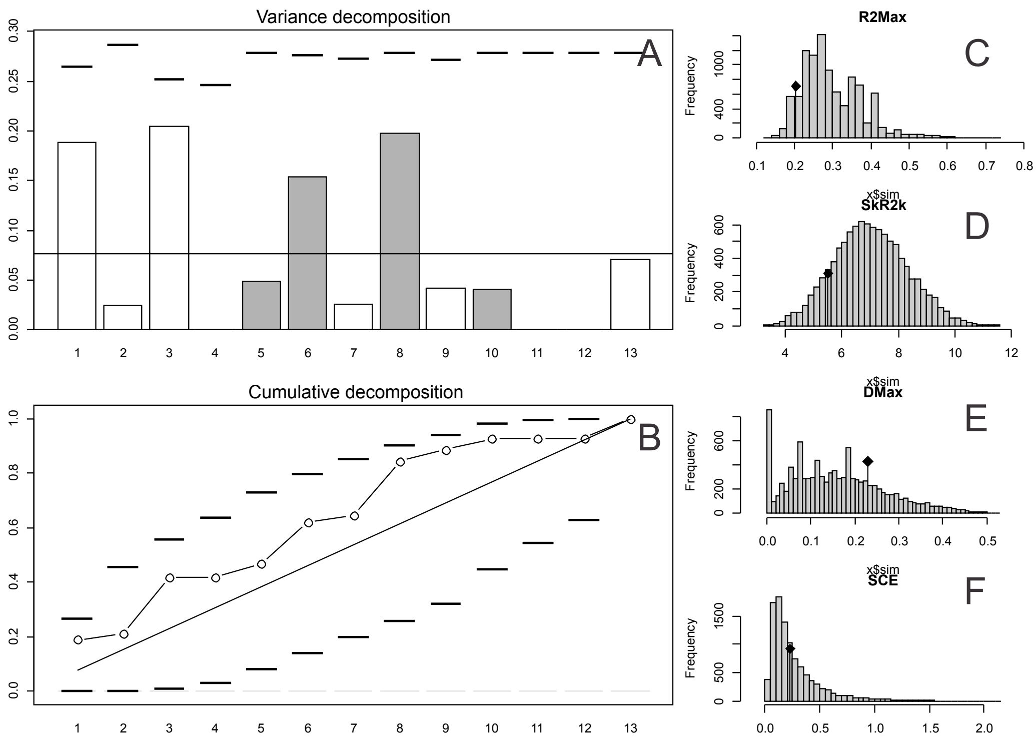

Fig. S10 - Orthonormal decomposition results of PC3 for Cladogram A. (A) Orthogram plot: height of bars is proportional to the squared coefficients (white and grey bars represents positive and negative coefficients); dashed line is the upper confidence limit at 5 %, built from Monte Carlo permutations; horizontal solid line is the mean value; (B) Cumulative orthogram plot: circles represent observed values of cumulated squared coefficients (vertical axis); the expected values under H0 are disposed on the straight line; dashed lines represent the bilateral confidence interval; (C–F) Histograms of observed values of the four statistic tests: black dot depicts the observed parameter value.

Non-parametric tests for Orthonormal decomposition

	Test	Obs	Std.Obs	Alter	Pvalue
1	R2Max	0.2046833	-1.1949816	greater	0.9142
2	SkR2k	5.5062188	-1.1635360	less	0.1232
3	Dmax	0.2296349	0.6611392	two-sided	0.5460
4	SCE	0.2307788	-0.1194382	greater	0.3993

Most significant orthobases

3 8 1 6 13 5 9 10 7 2

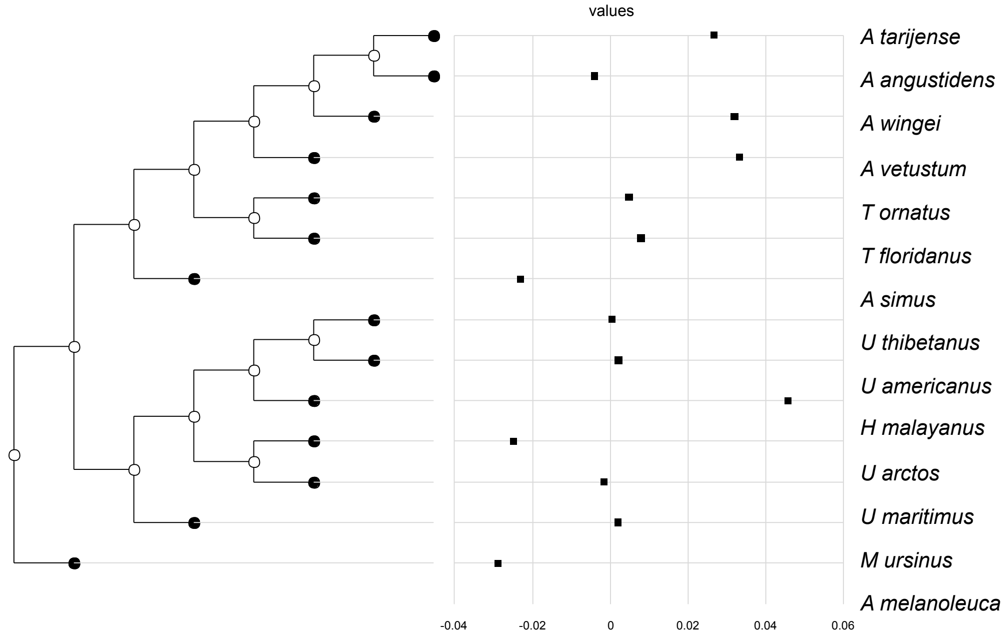

Dotplot of PC3 for cladogram A

Orthonormal variance decomposition results for cladogram B (PC3)

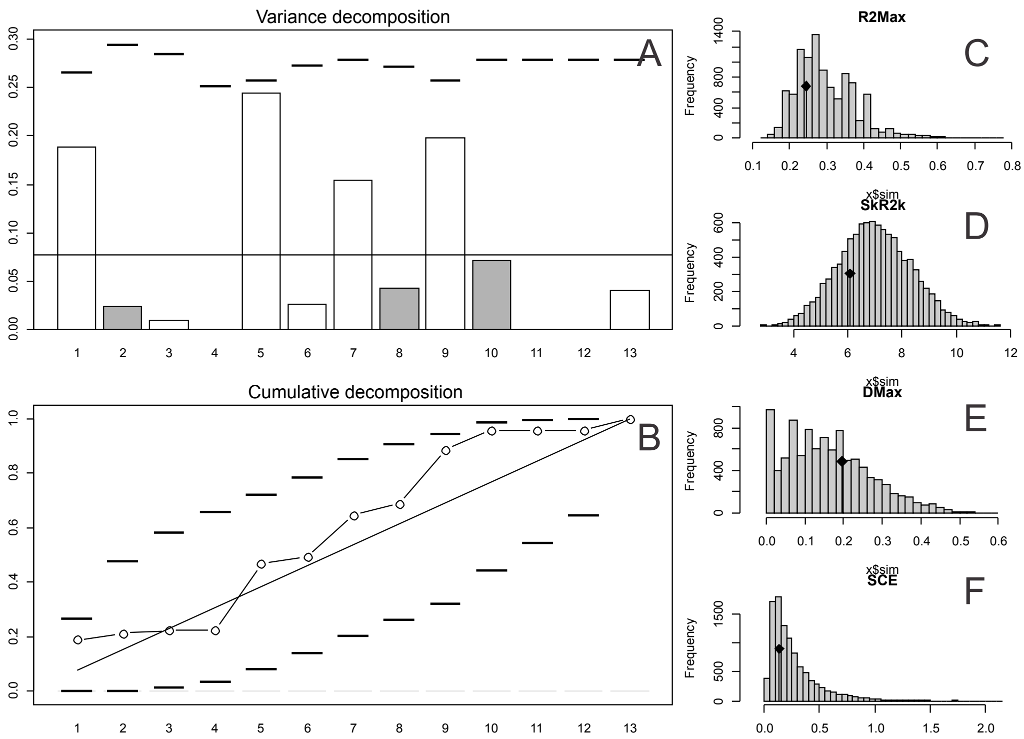

Fig. S11 - Orthonormal decomposition results of PC3 for Cladogram B. (A) Orthogram plot: height of bars is proportional to the squared coefficients (white and grey bars represents positive and negative coefficients); dashed line is the upper confidence limit at 5 %, built from Monte Carlo permutations; horizontal solid line is the mean value; (B) Cumulative orthogram plot: circles represent observed values of cumulated squared coefficients (vertical axis); the expected values under H0 are disposed on the straight line; dashed lines represent the bilateral confidence interval; (C–F) Histograms of observed values of the four statistic tests: black dot depicts the observed parameter value.

Non-parametric tests for Orthonormal decomposition

	Test	Obs	Std.Obs	Alter	Pvalue
1	R2Max	0.2442065	-0.6890774	greater	0.7142
2	SkR2k	6.0879188	-0.6928333	less	0.2506
3	Dmax	0.1951011	0.3149200	two-sided	0.7623
4	SCE	0.1366338	-0.5568426	greater	0.6544

Most significant orthobases

5 9 1 7 10 8 13 6 2 3

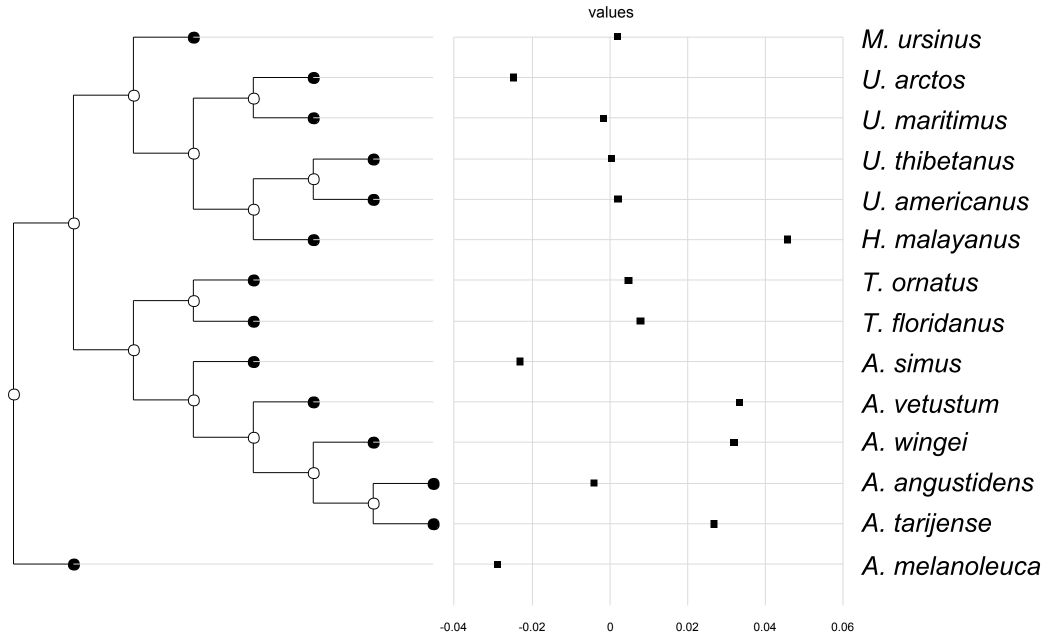

Dotplot of PC3 for cladogram B

Orthonormal variance decomposition results for cladogram A (PC4)

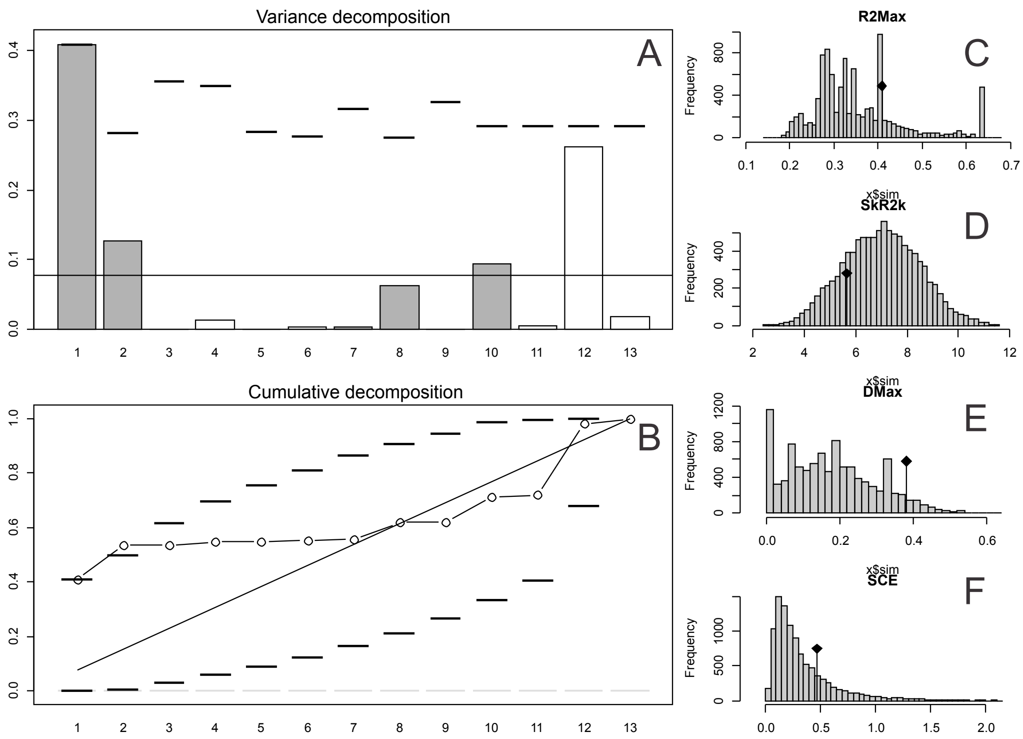

Fig. S12 - Orthonormal decomposition results of PC4 for Cladogram A. (A) Orthogram plot: height of bars is proportional to the squared coefficients (white and grey bars represents positive and negative coefficients); dashed line is the upper confidence limit at 5 %, built from Monte Carlo permutations; horizontal solid line is the mean value; (B) Cumulative orthogram plot: circles represent observed values of cumulated squared coefficients (vertical axis); the expected values under H0 are disposed on the straight line; dashed lines represent the bilateral confidence interval; (C–F) Histograms of observed values of the four statistic tests: black dot depicts the observed parameter value.

Non-parametric tests for Orthonormal decomposition

	Test	Obs	Std.Obs	Alter	Pvalue
1	R2Max	0.4089349	0.5316140	greater	0.2630
2	SkR2k	5.6589199	-0.9153509	less	0.1901
3	Dmax	0.3813561	1.6784057	two-sided	0.0561
4	SCE	0.4700824	0.5019569	greater	0.2088

Most significant orthobases

1 12 2 10 8 13 4 11 6 7

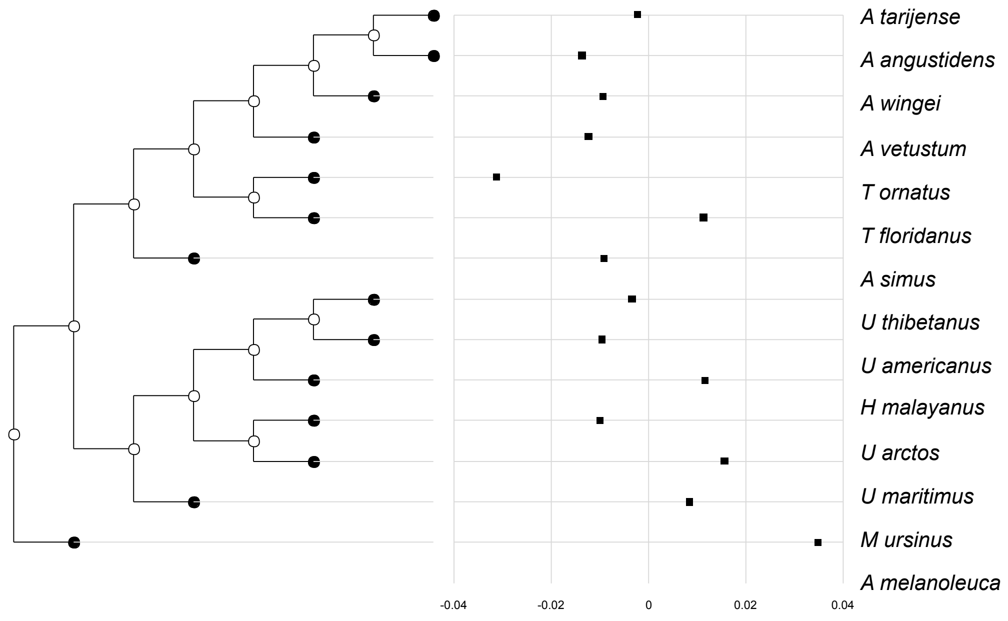

Dotplot of PC4 for cladogram A

Orthonormal variance decomposition results for cladogram B (PC4)

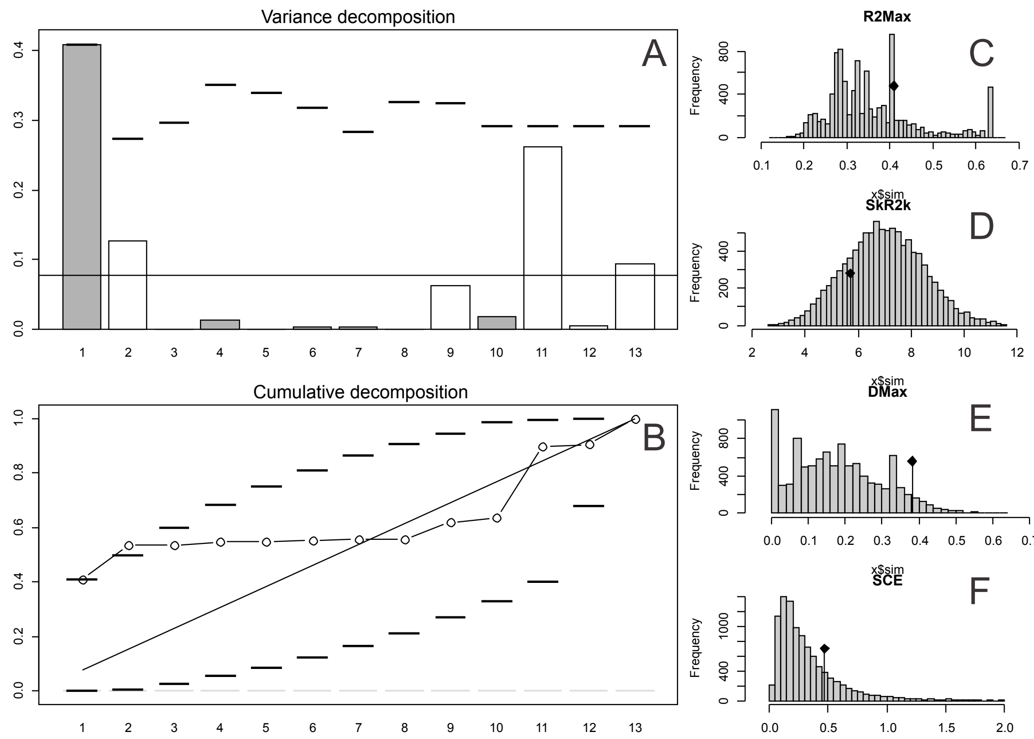

Fig. S13 - Orthonormal decomposition results of PC4 for Cladogram B. (A) Orthogram plot: height of bars is proportional to the squared coefficients (white and grey bars represents positive and negative coefficients); dashed line is the upper confidence limit at 5 %, built from Monte Carlo permutations; horizontal solid line is the mean value; (B) Cumulative orthogram plot: circles represent observed values of cumulated squared coefficients (vertical axis); the expected values under H0 are disposed on the straight line; dashed lines represent the bilateral confidence interval; (C–F) Histograms of observed values of the four statistic tests: black dot depicts the observed parameter value.

Non-parametric tests for Orthonormal decomposition

	Test	Obs	Std.Obs	Alter	Pvalue
1	R2Max	0.4089349	0.5047159	greater	0.2266
2	SkR2k	5.6940874	-0.8865282	less	0.1946
3	Dmax	0.3813561	1.6814854	two-sided	0.0546
4	SCE	0.4714768	0.5162238	greater	0.2073

Most significant orthobases

1 11 2 13 9 10 4 12 7 6

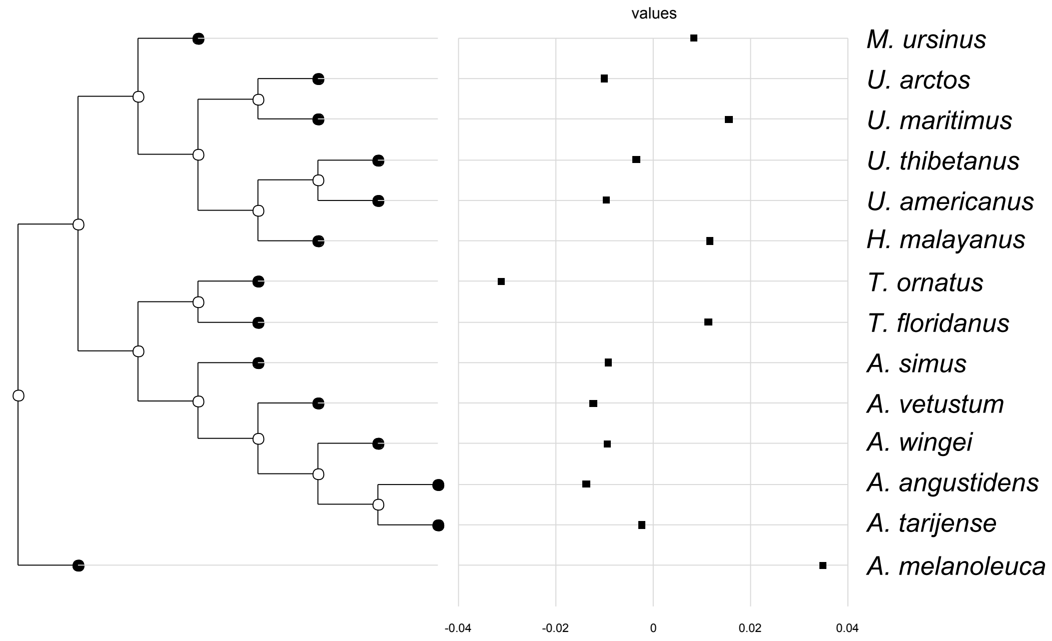

Dotplot of PC4 for cladogram B

Orthonormal variance decomposition results for cladogram A (PC5)

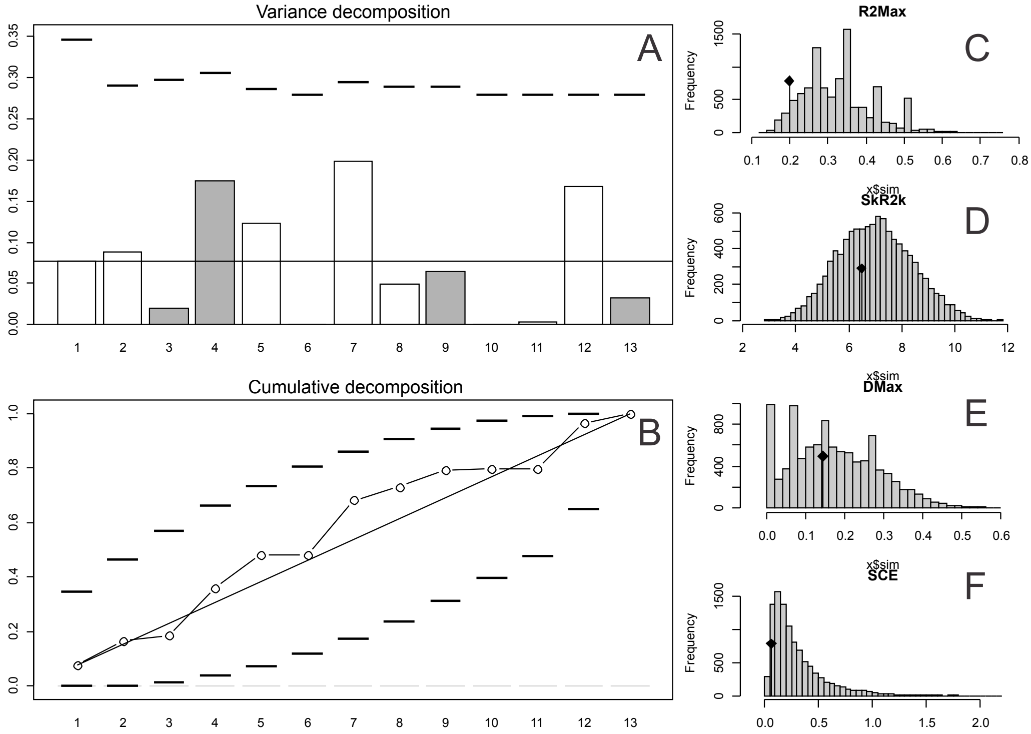

Fig. S14 - Orthonormal decomposition results of PC5 for Cladogram A. (A) Orthogram plot: height of bars is proportional to the squared coefficients (white and grey bars represents positive and negative coefficients); dashed line is the upper confidence limit at 5 %, built from Monte Carlo permutations; horizontal solid line is the mean value; (B) Cumulative orthogram plot: circles represent observed values of cumulated squared coefficients (vertical axis); the expected values under H0 are disposed on the straight line; dashed lines represent the bilateral confidence interval; (C–F) Histograms of observed values of the four statistic tests: black dot depicts the observed parameter value.

Non-parametric tests for Orthonormal decomposition

	Test	Obs	Std.Obs	Alter	Pvalue
1	R2Max	0.19874968	-1.4329946	greater	0.9499
2	SkR2k	6.47409912	-0.3695330	less	0.3709
3	Dmax	0.14374739	-0.2341769	two-sided	0.8463
4	SCE	0.06492226	-0.9417431	greater	0.9384

Most significant orthobases

7 4 12 5 2 1 9 8 13 3

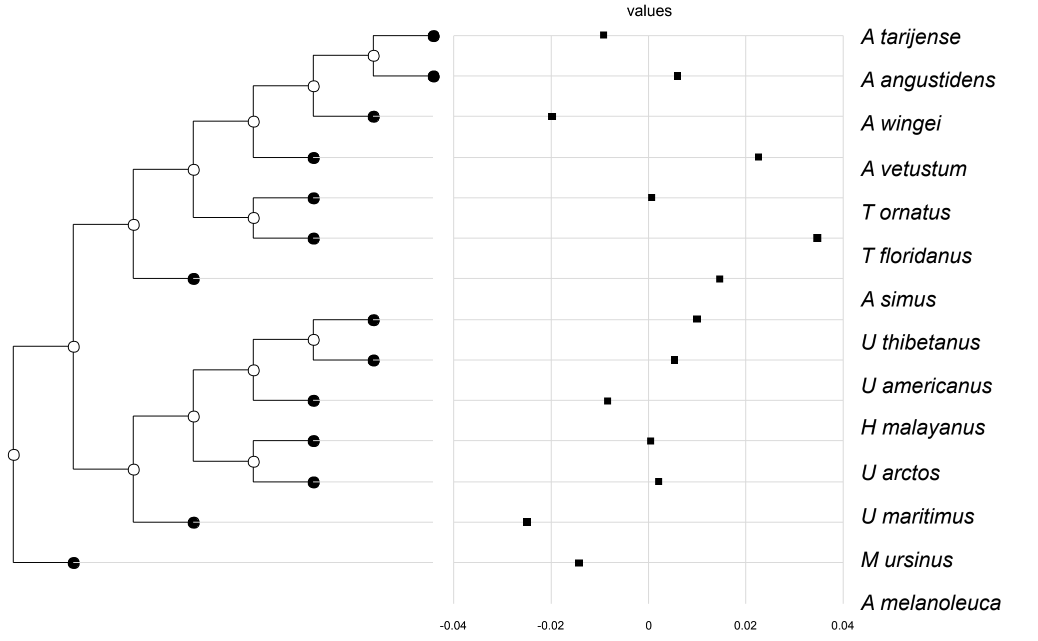

Dotplot of PC5 for cladogram A

Orthonormal variance decomposition results for cladogram B (PC5)

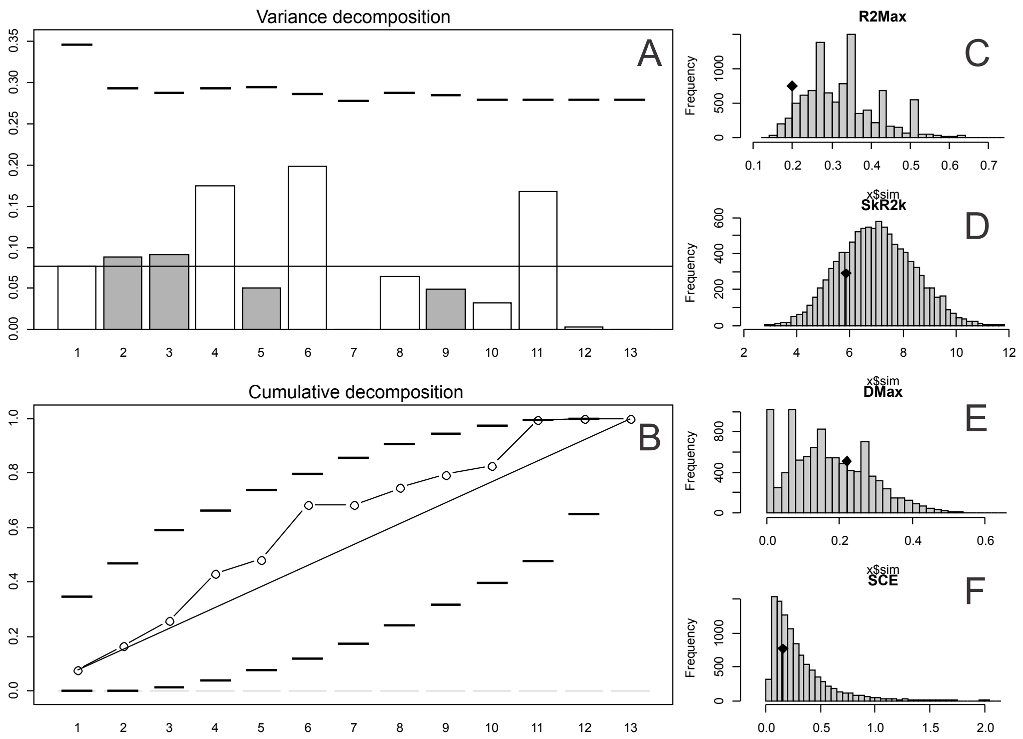

Fig. S15 - Orthonormal decomposition results of PC5 for Cladogram B. (A) Orthogram plot: height of bars is proportional to the squared coefficients (white and grey bars represents positive and negative coefficients); dashed line is the upper confidence limit at 5 %, built from Monte Carlo permutations; horizontal solid line is the mean value; (B) Cumulative orthogram plot: circles represent observed values of cumulated squared coefficients (vertical axis); the expected values under H0 are disposed on the straight line; dashed lines represent the bilateral confidence interval; (C–F) Histograms of observed values of the four statistic tests: black dot depicts the observed parameter value.

Non-parametric tests for Orthonormal decomposition

	Test	Obs	Std.Obs	Alter	Pvalue
1	R2Max	0.1987497	-1.4085965	greater	0.9502
2	SkR2k	5.8544935	-0.8280075	less	0.2139
3	Dmax	0.2203208	0.4675202	two-sided	0.6807
4	SCE	0.1549747	-0.5571463	greater	0.6550

Most significant orthobases

6 4 11 3 2 1 8 5 9 10

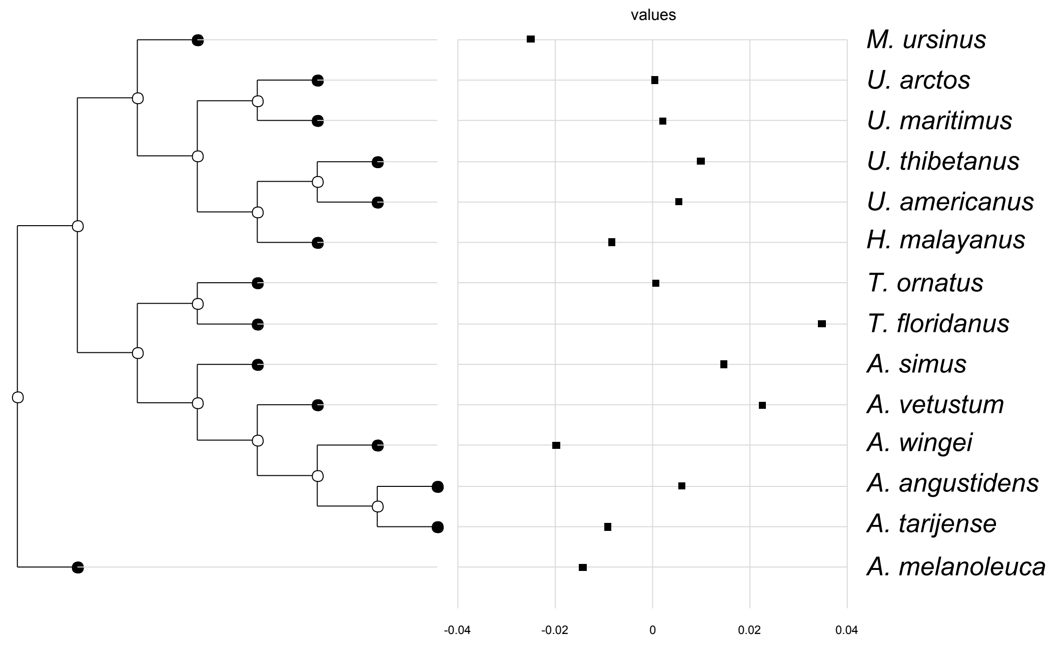

Dotplot of PC5 for cladogram B

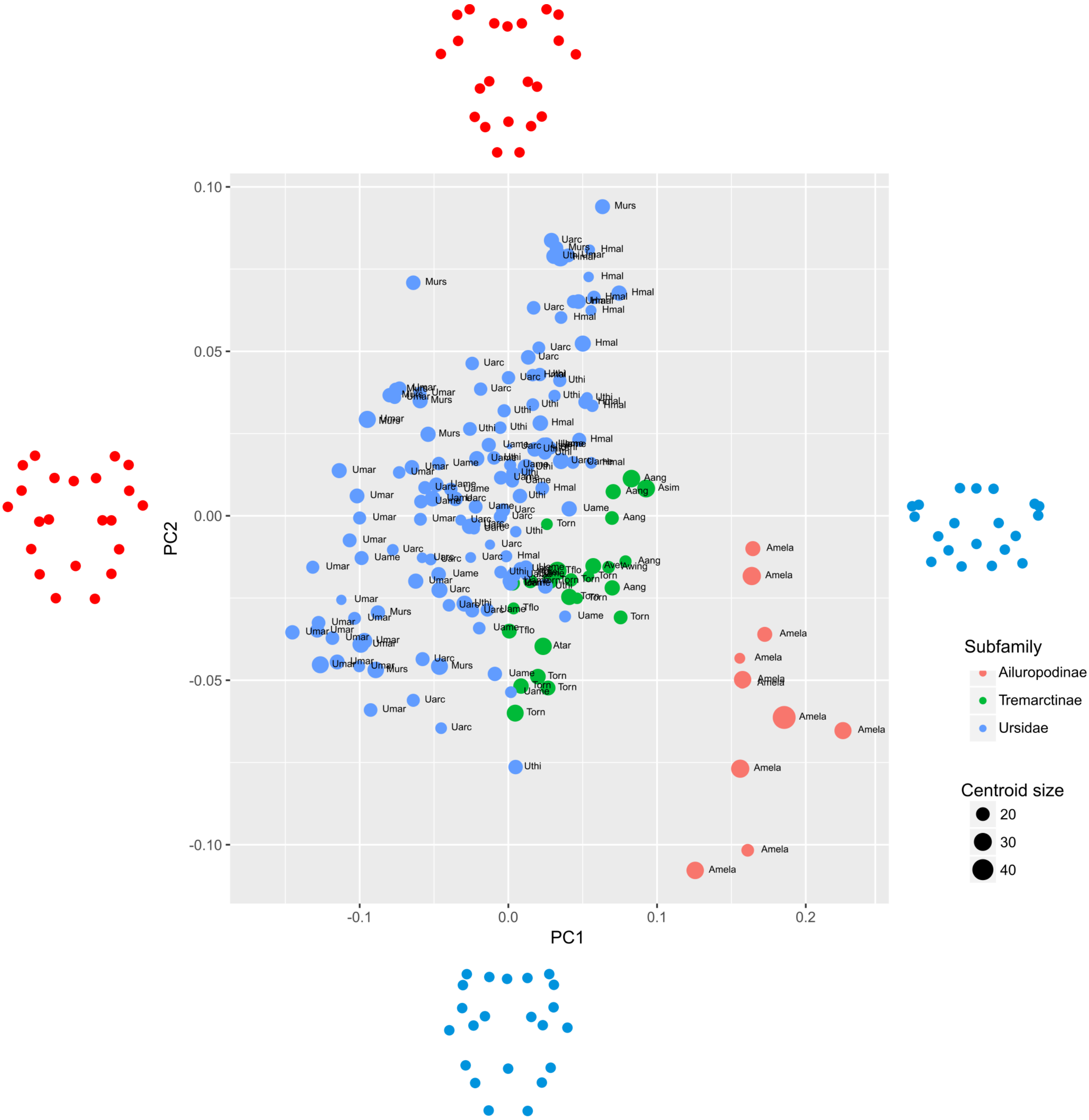

Figure S16- Scatterplot of the first two principal components (PC) of basicranium shape of Ursidae. Size of circles is scaled according to the Centroid Size. The shapes correspond to minimum and maximum landmark configurations for the PC1 (horizontal axis) and the minimum and maximum configurations for PC2 (vertical axis).
