# Supplementary material for: Phylogenetic signal analysis in the basicranium of Ursidae (Carnivora, Mammalia)
Source: PeerJ. 2019 Mar 15;7:e6597. doi: 10.7717/peerj.6597 (PMC6422017; doi:10.7717/peerj.6597)
Supplement: Table S1 — Abbreviations: F, female; M, male; NS, not sexed. [file peerj-07-6597-s004.rtf]

Table S1- List of the specimens used in the geometric morfometric analysis. Abbreviations: F: female; M: male; NS: not sexed.
Specimen	Taxon	Sex	Location	Subfamily	
MACN12529	A. angustidens	F	South America	Tremarctinae	
MLP00-VII-10-1	A. angustidens	M	South America	Tremarctinae	
MLP82-X-22-1	A. angustidens	F	South America	Tremarctinae	
MMP1491	A. angustidens	NS	South America	Tremarctinae	
MMP162S	A. angustidens	NS	South America	Tremarctinae	
AMNH99209	Ar. simus	NS	North America	Tremarctinae	
AMNH56264	T. floridanus	NS	North America	Tremarctinae	
AMNH98965	T. floridanus	NS	North America	Tremarctinae	
MMP1233	A. vetustum	NS	South America	Tremarctinae	
MMP971	A. tarijense	NS	South America	Tremarctinae	
GP-2T 04	A. wingei	NS	South America	Tremarctinae	
MLP MLP 1-I-03-62	T. ornatus	F	South America	Tremarctinae	
MLP 2329	T. ornatus	F	South America	Tremarctinae	
USNM 100657	T. ornatus	F	South America	Tremarctinae	
USNM155575	T. ornatus	NS	South America	Tremarctinae	
USNM170656	T. ornatus	F	South America	Tremarctinae	
USNM194309	T. ornatus	F	South America	Tremarctinae	
USNM210323	T. ornatus	M	South America	Tremarctinae	
USNM271418	T. ornatus	M	South America	Tremarctinae	
USNM271419	T. ornatus	NS	South America	Tremarctinae	
USNM271420	T. ornatus	M	South America	Tremarctinae	
USNM282620	T .ornatus	F	South America	Tremarctinae	
USNM582002	T. ornatus	F	South America	Tremarctinae	
AMNH104758	U. vitavilis	NS	North America	Ursinae	
AMNH30780	U. arctos	NS	North America	Ursinae	
AMNH95595	U. arctos	NS	North America	Ursinae	
CA07707	U. arctos	NS	North America	Ursinae	
ETVP5587	U. arctos	NS	North America	Ursinae	
USNM 181100 	U. arctos	M	North America	Ursinae	
USNM 201586	U. arctos	M	North America	Ursinae	
USNM 203284	U. arctos	M	North America	Ursinae	
USNM 203290	U. arctos	M	North America	Ursinae	
USNM 203526 	U. arctos	M	North America	Ursinae	
USNM 211460 	U. arctos	M	North America	Ursinae	
USNM 211677	U. arctos	M	North America	Ursinae	
USNM 212817 	U. arctos	M	North America	Ursinae	
USNM 213007 	U. arctos	M	North America	Ursinae	
USNM 213698 	U. arctos	F	North America	Ursinae	
USNM 213698 	U. arctos	F	North America	Ursinae	
USNM 215453	U. arctos	M	North America	Ursinae	
USNM 215456	U. arctos	F	North America	Ursinae	
USNM 221614	U. arctos	F	North America	Ursinae	
USNM 221627	U. arctos	F	North America	Ursinae	
USNM 222493	U. arctos	F	North America	Ursinae	
USNM 222494	U. arctos	F	North America	Ursinae	
USNM 224801	U. arctos	F	North America	Ursinae	
USNM 227847	U. arctos	M	North America	Ursinae	
USNM 228095	U. arctos	F	North America	Ursinae	
USNM 234240	U. arctos	F	North America	Ursinae	
USNM 235098	U. arctos	F	North America	Ursinae	
USNM 242261	U. arctos	F	North America	Ursinae	
USNM 242262	U. arctos	M	North America	Ursinae	
AMNH42791	U. americanus	NS	North America	Ursinae	
USNM 08705 	U. americanus	F	North America	Ursinae	
USNM 08706 	U. americanus	F	North America	Ursinae	
USNM 138681 	U. americanus	F	North America	Ursinae	
USNM 156597 	U. americanus	M	North America	Ursinae	
USNM 159337 	U. americanus	F	North America	Ursinae	
USNM 170868 	U. americanus	F	North America	Ursinae	
USNM 174801 	U. americanus	NS	North America	Ursinae	
USNM 174803	U. americanus	NS	North America	Ursinae	
USNM 211749	U. americanus	M	North America	Ursinae	
USNM 215134	U. americanus	M	North America	Ursinae	
USNM 215202	U. americanus	F	North America	Ursinae	
USNM 216420 	U. americanus	M	North America	Ursinae	
USNM 223500 	U. americanus	F	North America	Ursinae	
USNM 224509	U. americanus	F	North America	Ursinae	
USNM 224537	U. americanus	F	North America	Ursinae	
USNM 227660	U. americanus	M	North America	Ursinae	
USNM 230788	U. americanus	F	North America	Ursinae	
USNM 232488	U. americanus	F	North America	Ursinae	
USNM 243982	U. americanus	F	North America	Ursinae	
USNM 247319 	U. americanus	F	North America	Ursinae	
USNM080704	U. americanus	M	North America	Ursinae	
USNM135141	U. americanus	M	North America	Ursinae	
USNM135322	U. americanus	M	North America	Ursinae	
USNM146371 	U. americanus	M	North America	Ursinae	
USNM150643	U. americanus	M	North America	Ursinae	
USNM157339	U. americanus	M	North America	Ursinae	
USNM170869	U. americanus	M	North America	Ursinae	
USNM234627	U. americanus	M	North America	Ursinae	
USNM234628	U. americanus	M	North America	Ursinae	
CA RB0909	M. ursinus	NS	Asia	Ursinae	
CA RB2899	M. ursinus	NS	Asia	Ursinae	
USNM 151532	M. ursinus	NS	Asia	Ursinae	
USNM125506	M. ursinus	F	Asia	Ursinae	
USNM199518	M. ursinus	F	Asia	Ursinae	
USNM253300	M. ursinus	NS	Asia	Ursinae	
USNM253301	M. ursinus	NS	Asia	Ursinae	
USNM253302	M. ursinus	NS	Asia	Ursinae	
USNM535017	M. ursinus	M	Asia	Ursinae	
USNM581890	M. ursinus	F	Asia	Ursinae	
USNM232553	M. ursinus	NS	Asia	Ursinae	
USNM 021845	U. thibetanus	M	Asia	Ursinae	
USNM 187866	U. thibetanus	M	Asia	Ursinae	
USNM 199684	U. thibetanus	F	Asia	Ursinae	
USNM 218152 	U. thibetanus	M	Asia	Ursinae	
USNM 240670	U. thibetanus	F	Asia	Ursinae	
USNM 2506069	U. thibetanus	F	Asia	Ursinae	
USNM 258647	U. thibetanus	F	Asia	Ursinae	
USNM 259099	U. thibetanus	F	Asia	Ursinae	
USNM 271090	U. thibetanus	M	Asia	Ursinae	
USNM083453	U. thibetanus	NS	Asia	Ursinae	
USNM084092	U. thibetanus	F	Asia	Ursinae	
USNM084093	U. thibetanus	M	Asia	Ursinae	
USNM258430	U. thibetanus	NS	Asia	Ursinae	
USNM258546	U. thibetanus	M	Asia	Ursinae	
USNM258593	U. thibetanus	M	Asia	Ursinae	
USNM258645	U. thibetanus	M	Asia	Ursinae	
USNMA22998	U. thibetanus	NS	Asia	Ursinae	
USNM 174801	U. maritimus	NS	North America	Ursinae	
USNM 174803	U. maritimus	NS	North America	Ursinae	
USNM 215149	U. maritimus	F	North America	Ursinae	
USNM 227096	U. maritimus	NS	North America	Ursinae	
USNM 227111	U. maritimus	NS	North America	Ursinae	
USNM 228309	U. maritimus	F	North America	Ursinae	
USNM 512103	U. maritimus	F	North America	Ursinae	
USNM 512105	U. maritimus	F	North America	Ursinae	
USNM 512117	U. maritimus	M	North America	Ursinae	
USNM 512124	U. maritimus	F	North America	Ursinae	
USNM 512129	U. maritimus	NS	North America	Ursinae	
USNM 512134	U. maritimus	M	North America	Ursinae	
USNM 512143	U. maritimus	F	North America	Ursinae	
USNM 512151	U. maritimus	F	North America	Ursinae	
USNM 512157	U. maritimus	F	North America	Ursinae	
USNM082002	U. maritimus	M	North America	Ursinae	
USNM221000	U. maritimus	M	North America	Ursinae	
USNM227099	U. maritimus	M	North America	Ursinae	
USNM227104	U. maritimus	M	North America	Ursinae	
USNM227105	U. maritimus	M	North America	Ursinae	
USNM227107	U. maritimus	M	North America	Ursinae	
USNM291756	U. maritimus	NS	North America	Ursinae	
USNM511225	U. maritimus	M	North America	Ursinae	
USNM512112	U. maritimus	M	North America	Ursinae	
USNM512116	U. maritimus	F	North America	Ursinae	
USNM	U. maritimus	F	North America	Ursinae	
USNM19206	H. malayanus	M	Asia	Ursinae	
USNM115695	H. malayanus	M	Asia	Ursinae	
USNM123139	H. malayanus	F	Asia	Ursinae	
USNM142344	H. malayanus	NS	Asia	Ursinae	
USNM151866	H. malayanus	M	Asia	Ursinae	
USNM153835	H. malayanus	M	Asia	Ursinae	
USNM153838	H. malayanus	NS	Asia	Ursinae	
USNM153839	H. malayanus	NS	Asia	Ursinae	
USNM197254	H. malayanus	F	Asia	Ursinae	
USNM197255	H. malayanus	F	Asia	Ursinae	
USNM198713	H. malayanus	M	Asia	Ursinae	
USNM198714	H. malayanus	F	Asia	Ursinae	
USNM198715	H. malayanus	F	Asia	Ursinae	
USNM239451	H. malayanus	F	Asia	Ursinae	
USNM267586	H. malayanus	NS	Asia	Ursinae	
USNM358645	H. malayanus	NS	Asia	Ursinae	
USNM395845	H. malayanus	F	Asia	Ursinae	
USNM399301	H. malayanus	F	Asia	Ursinae	
USNM538095	H. malayanus	M	Asia	Ursinae	
USNM 258423	Ai. melanoleuca	NS	Asia	Ailuropodinae	
USNM 258836	Ai. melanoleuca	M	Asia	Ailuropodinae	
USNM 259027	Ai. melanoleuca	M	Asia	Ailuropodinae	
USNM 259029	Ai. melanoleuca	M	Asia	Ailuropodinae	
USNM 259074	Ai. melanoleuca	M	Asia	Ailuropodinae	
USNM 259076	Ai. melanoleuca	M	Asia	Ailuropodinae	
USNM 259402	Ai. melanoleuca	M	Asia	Ailuropodinae	
USNM258834	Ai. melanoleuca	M	Asia	Ailuropodinae	
USNM259400	Ai. melanoleuca	F	Asia	Ailuropodinae	
USNM399447	Ai. melanoleuca	NS	Asia	Ailuropodinae	
USNMA259401	Ai. melanoleuca	M	Asia	Ailuropodinae	
