# Supplementary material for: Phylogenetic signal analysis in the basicranium of Ursidae (Carnivora, Mammalia)
Source: PeerJ. 2019 Mar 15;7:e6597. doi: 10.7717/peerj.6597 (PMC6422017; doi:10.7717/peerj.6597)
Supplement: Table S3 [file peerj-07-6597-s006.rtf]

Table S3- Non-parametrical test for differences in skull ratio between subfamilies in this study.
Kruskal-Wallis rank sum test	
chi-squared	df	p-value	
42.655	2	0.0000	
	
Pairwise comparisons using Wilcoxon rank sum test	
p-value	Ailuropodinae	Tremarctinae	Ursinae	
Ailuropodinae	-			
Tremarctinae	0.00001	-		
Ursinae	0.00000	0.00016	-	
