# Supplementary material for: Phylogenetic signal analysis in the basicranium of Ursidae (Carnivora, Mammalia)
Source: PeerJ. 2019 Mar 15;7:e6597. doi: 10.7717/peerj.6597 (PMC6422017; doi:10.7717/peerj.6597)
Supplement: Table S4 [file peerj-07-6597-s007.rtf]

Table S4- Summary of the habitat, diet, weight and hibernation/dormancy of Ursinae bears 
Taxon	Habitat	Diet	Weight (Kg)	Hibernation/dormancy 	
Helarctos malayanus	Dense forest at all elevations. Climbs well	Wild honey, insects, larvae, coconut palm, birds, small rodents and fruits	27-65	no	
Melursus ursinus	Moist and dry forest especially in rocky outcrops	Termites	55-145 	no	
Ursus thibetanus	Moist deciduous forest, especially in the hills and mountains. Swims and climbs well.	Fruits, buds, invertebrates, small vertebrates and carrion	50-110	no	
Ursus americanus	Forested areas. Swims and climbs well	75% vegetable fruits, nuts, berries, acorns, grass and roots. Also fish, insects, rodents, carrion, occasionally large mammals	92-270 	At least in some areas	
Ursus arctos	Open areas but needs dense cover to shelter.	Mainly vegetation, also Fish, berries, small and large mammals.	150-780 	yes	
Ursus maritimus	Open areas	Seals, birds, large mammals, vegetation	150-800 	yes	
Tremarctos ornatus	Humid forest. Climbs well	fruits, leaves, opportunistic scavenge, rodents, small deer	60-170 	no	
Arctodus	Open areas	Omnivore/carnivore
Mainly carnivore?	300-800 	?	
Arctotherium	Open areas	Omnivore/carnivore
Carnivores with a tendency to hipocarnivory	150-1200	 at least in A. angustidens	
