# Supplementary material for: Phylogenetic signal analysis in the basicranium of Ursidae (Carnivora, Mammalia)
Source: PeerJ. 2019 Mar 15;7:e6597. doi: 10.7717/peerj.6597 (PMC6422017; doi:10.7717/peerj.6597)
Supplement: Table S5 [file peerj-07-6597-s008.rtf]

Table S5- Average of the skull ratio of the Tremarctinae species included in this study. 
Specie	Skull ratio	
Arctotherium angustidens	0.203522	
Arctodus simus	0.192025	
Arctotherium vetustum	0.237235	
Arctotherium tarijense	0.243578	
Arctotherium wingei	0.268803	
Tremarctos floridanus	0.245722	
Tremarctos ornatus	0.22702	
